# Integrated analysis of tumor and adjacent non-tumor proteomic data reveals SERPINH1 as a recurrence biomarker and drug target in hepatocellular carcinoma

**DOI:** 10.7150/ijbs.99734

**Published:** 2024-09-23

**Authors:** Yushan Hou, Yiming Zhang, Kun Zheng, Han Wang, Yingying Zhou, Yuanjun Zhai, Fuchu He, Chunyan Tian, Aihua Sun

**Affiliations:** 1 State Key Laboratory of Medical Proteomics, National Center for Protein Sciences (Beijing), Proteome Research Center, Beijing Institute of Lifeomics, Beijing, China.; 2 Department of Orthopedics, General Hospital of Southern Theater Command, Guangzhou, China.; 3 Department of Pathology, Shanghai Eastern Hepatobiliary Surgery Hospital, Naval Medical University, Shanghai, China.; 4 Research Unit of Proteomics Dirven Cancer Precision Medicine, Chinese Academy of Medical Sciences, Beijing, China.

**Keywords:** non-tumor adjacent tissues, recurrence, biomarker, SERPINH1, Col003

## Abstract

The high rate of postoperative recurrence contributes to the poor outcome in hepatocellular carcinoma (HCC), and effective strategies for managing recurrence are currently lacking. Based on seven pairs of tumors and non-tumor adjacent tissues (NATs) proteomic datasets across five cancer types, this study systematically investigates the stratified and therapeutic value of tumors and NATs for tumor recurrence. NATs exhibited stable and irreplaceable independent prognostic capabilities for recurrence, complementing clinical indicators and tumor characteristics. In comparison to tumor tissues, NATs exhibit higher enrichment levels of recurrence-related proteins in pathways such as immunity, extracellular matrix, and angiogenesis. Taking HCC as an example, we identified SERPINH1 as a recurrent biomarker with drug-targeting potential that applied to both tumors and NATs and then validated them through independent immunohistochemistry cohorts and animal experiments. Patients with high SERPINH1 expression in both tumors and NATs have the highest 5-year recurrence rates, even among clinically low recurrence risk groups. Targeting SERPINH1 can effectively delay tumor occurrence and progression. This study highlights the significant importance of NATs in recurrence prediction and postoperative management, proposing a recurrence management strategy that focuses on both tumors and NATs. SERPINH1 emerges as a valuable biomarker and drug target for addressing postoperative recurrence in HCC.

## Background

Hepatocellular carcinoma (HCC) is one of the deadliest malignancies worldwide, with a survival rate of only 22%. It is estimated that there will be 865,269 new cases of HCC and 757,948 HCC-related deaths in 2024 1, 2. Surgical resection is the primary treatment for HCC. However, its major drawback is the high postoperative recurrence rate of up to 80% and there are no recognized adjuvant therapy methods available 3. Identifying high-risk populations of postoperative recurrence and providing individualized treatment can effectively improve overall survival rates of HCC.

Currently, targeted drug research mainly focuses on patients with advanced-stage HCC unsuitable for surgical resection, such as sorafenib, lenvatinib, and immune checkpoint inhibitors. These therapies focus on reducing tumor burden and prolonging the survival time of patients 4, 5. Therefore, the majority of clinical and basic studies on HCC biomarkers and targets continue to focus on tumor tissues. Clinicians have come to recognize certain features, such as microvascular invasion (MVI), serum alpha-fetoprotein (AFP) levels exceeding 400 μg/L, multifocality, tumor diameter greater than 5 cm, as well as the presence of hepatitis and cirrhosis, all indicating an increased risk of postoperative recurrence in HCC patients 6, 7. In addition, the presence of circulating tumor cells (CTCs), circulating tumor DNA (ctDNA), and changes in the abundance of some proteins (CXCL10, ASPH, SPP2, ENO2, etc) all predicted the risk of postoperative recurrence 8-12. Proteomic subtype analysis of tumor tissues has identified molecular subtypes with prognostic differences, and highlighted genes such as SOAT1, PYCR2, and ADH1A as prognostic markers and therapeutic targets, opening new avenues for precision therapy in HCC 13-15.

Unlike patients with advanced-stage HCC who have long-term tumor burden, postoperative HCC patients lack targetable tumor tissue over a long period. Therefore, the management of postoperative recurrence differs from that of advanced-stage HCC patients. Patients with MVI-positive, multifocal tumors were defined clinically as high-risk recurrence patients, while patients with single foci without MVI but with tumor diameters greater than 5 cm were defined as medium-risk recurrence patients. Both of them were recommended to undergo postoperative transarterial chemoembolization (TACE) treatment 16. However, this standard does not account for the fact that 21.9% of low-risk patients with MVI negativity, single lesions, and tumor diameters smaller than 5 cm experience relapse within two years, with a median recurrence-free survival of only 29 months. Furthermore, TACE therapy targeting the tumor itself does not lead to improved outcomes in low-risk populations 17. Consequently, researchers have begun to investigate factors beyond the tumor that may impact patient prognosis. According to the "seed and soil" theory, both the malignant phenotype of the tumor cells and the corresponding microenvironment contribute to recurrence 18, 19. Therefore, NAT has garnered growing attention as the "soil" for tumor development. Analysis of gene expression profiles of NAT in HCV-dominant HCC cohorts has demonstrated that peritumoral gene expression characteristics can reliably predict long-term survival and late recurrence (>2 years), surpassing the predictive ability of tumor tissue 20. Similarly, gene expression chip analysis of HCV-related HCC has confirmed the superior efficacy of NAT in predicting late recurrence events (>1 year) compared to tumor tissue 21. NATs in HCC can be divided into three prognostically different subtypes 22. The immunologic and angiogenic status of NATs is closely associated with the postoperative recurrence rate and overall survival of patients 23, 24.

Additionally, research has shown that drugs targeting immunity and angiogenesis, which simultaneously regulate the tumor and its peri-tumor microenvironment, such as sintilimab, atezolizumab combined with bevacizumab can significantly prolong the recurrence-free survival of patients after surgery 25, 26. However, these results need to be confirmed by multicenter, multinational phase 3 trials, and long-term follow-up. Hence, the systematic analysis of NATs' role in tumorigenesis and development is anticipated to lead to the discovery of novel prognostic markers and intervention targets, ultimately enhancing the prognosis of HCC patients.

Accordingly, we collected seven pairs of tumor and NAT proteomic datasets from five solid tumor types to systematically elucidate the predictive value and primary functions of tumors and NATs in tumor recurrence 13-15, 27-30. The study revealed that the number and predictive efficacy of recurrence-related proteins in NATs are similar to those found in tumor tissue. Additionally, it was observed that NAT recurrence features consistently demonstrate stable predictive capabilities across the seven datasets. Compared to tumor tissues, recurrence-related proteins in NATs exhibit higher enrichment levels in pathways related to immune response, extracellular matrix, and angiogenesis. On the other hand, tumor tissue tends to focus on functions associated with proliferation-related functions such as alternative splicing, ribosome biogenesis, and cell cycle regulation. More importantly, the recurrence features of NAT complement various clinical indicators and tumor molecular features and are irreplaceable. Subsequently, taking HCC as an example, SERPINH1 was screened as a biomarker and drug target capable of predicting prognosis in both tumors and NATs simultaneously. The predictive ability of its recurrence was validated through immunohistochemistry in both the overall population and low-risk relapse population defined by clinical indicators. Further, an *in vivo* mouse experiment demonstrated that targeting SERPINH1 significantly enhanced the prognosis of HCC.

## Methods

### Proteomic data processing and DFS subtype definition

The proteomic data were normalized with quantile normalization using the R package limma v.3.50.3, and missing values were imputed with the minimum value across the data. In addition, proteomic data produced by experiments were performed using a centered log-ratio transformation before UMAP. We used the R package Survminer v0.4.9 to determine the best cut-off for disease-free/overall survival and calculate the log-rank p values and the hazard ratios (HR). Proteins with p<0.05 were identified as recurrence-related proteins (RRPs). If the HR is greater than 1, it is defined as unfavorable. Otherwise, it is defined as favorable. We screened unfavorable and favorable proteins in tumors and NATs to form four gene sets. Single sample gene set enrichment analysis (ssGSEA) was performed to calculate separate enrichment scores(ES) of 4 gene sets for each sample by the R package GSVA v1.42.0. DFS score is defined as ES_unfavor_ minus ES_favor_. We divided the subtypes according to the best cut-off of the tumor and adjacent non-tumor DFS scores given by Survminer, respectively.

### Functional enrichment analysis

Enrichment analyses of gene ontology (GO), Reactome, and Kyoto Encyclopedia of Genes and Genomes (KEGG) pathway were conducted on the RRPs by using the R package clusterProfiler v4.2.2. In HCC, the target set is selected from proteins that are significantly unfavorable in at least one cohort and are not significantly favorable in any cohorts. The foldchange of differentially expressed genes between groups defined by RRP was calculated using the R package limma. Gene set enrichment analysis (GSEA) was performed on all genes. GSEA was run with Molecular Signatures Database (MSigDB) set v7.4, C2: curated gene sets (browse 6290 gene sets), C5: Gene Ontology gene sets (browse 10461 gene sets) using the R package GSVA v1.42.0. P-value less than 0.05 was considered significant in all the analyses.

### Validation cohort and immunohistochemistry (IHC)

Tissue microarray (TMA) of 79 HCC patients (IHC cohort1) for IHC analysis were acquired from the Shanghai Xinchao Biotechnology Co. Ltd. TMA of 95 patients with hepatocellular carcinoma obtained from the Department of Pathology, Eastern Hepatobiliary Surgery Hospital in Shanghai (IHC cohort2). All patients in IHC cohort2 with only a solitary tumor lesion of less than or equivalent to 5cm and without microvascular invasion were previously assessed as low-risk for recurrence by the clinical risk.

All IHC for SERPINH1 were carried out using standard protocols on formalin-fixed, paraffin-embedded TMA sections. The antibody used was sc-5293 (Santa Cruz Biotechnology, 1:70). TMA was scored according to the percentage of positive stromal cells and stained intensity. The scoring criteria are as follows: 0%=0, [1,25%)=1, [25,50%)=2, [50,75%)=3, [75,100%)=4; no staining=0, weak staining=1, moderate staining=2, strong staining=3. The final score was determined by multiplying the intensity and extent of positive scores of stained cells, with a minimum score of 0 and a maximum score of 12. Tissue microarrays were scored by two pathologists independently.

### Animal study

Two-week-old C57BL/6J male mice obtained from Vital River Laboratory (Beijing, China) were injected intraperitoneally (i.p.) with DEN (25 mg/kg). Two weeks later, mice were injected i.p. weekly with CCl_4_ (0.5 ml/kg body weight, and lasting for 14 weeks). Ten weeks post the initial CCl_4_ treatment, mice used for the model were randomly assigned to separate groups and injected i.p. with either 10 mg/kg Col003 (MedChemExpress, #HY-124817) or corresponding vehicle twice weekly for 11 weeks. Mice were euthanized two weeks following the final Col003 administration to enable subsequent analyses.

The hematoxylin and eosin (H&E) and Sirius Red staining were performed by Bairuisi Biotechnology Co., Ltd (Tianjin, China). Mouse liver tissues were fixed in 4% paraformaldehyde, subsequently embedded in paraffin wax, and sectioned into 4-micrometer-thick slices. The sections underwent staining with H&E or Sirius Red and scanning for further analysis.

### Proteomics

Mice liver tissues were rapidly frozen with liquid nitrogen and lysed using SDC lysis buffer (100 mM Tris, pH 8.5, 1% DOC, 10 mM TCEP, 40 mM CAA) to extract proteins. The proteins quantified by the BCA Protein Assay were digested by trypsin overnight. The digested peptides were acidified by formic acid (FA) to a final FA concentration of 1% and then centrifuged at 16,000g for 10 min. The supernatants were desalted via a homemade C18 stage tip. The tip was activated with acetonitrile, equilibrated twice with 0.1% FA, and the supernatant was loaded. The column was washed twice with 0.1% FA, and peptides were eluted using 50% acetonitrile in 0.1% FA. The peptides were analyzed by MS using QE-HF for 120 min. The raw data underwent MaxQuant v 2.0.1.0-based database searching for protein identification and quantification.

### Statistics

The statistical analyses were performed using R software v4.1.2 and GraphPad Prism 9. Disease-free survival (DFS) curves were plotted according to the Kaplan-Meier method and compared using the log-rank test. The hazard ratios (HR) were computed with log-rank tests using univariate analysis based on a Cox proportional hazards regression model. Wilcoxon rank-sum test and chi-square test was used to compare differences between groups. P value < 0.05 was considered to indicate statistical significance.

## Results

### The NATs possess prognostic predictive abilities comparable to tumors, albeit with different functional emphases

**Figure 1:**
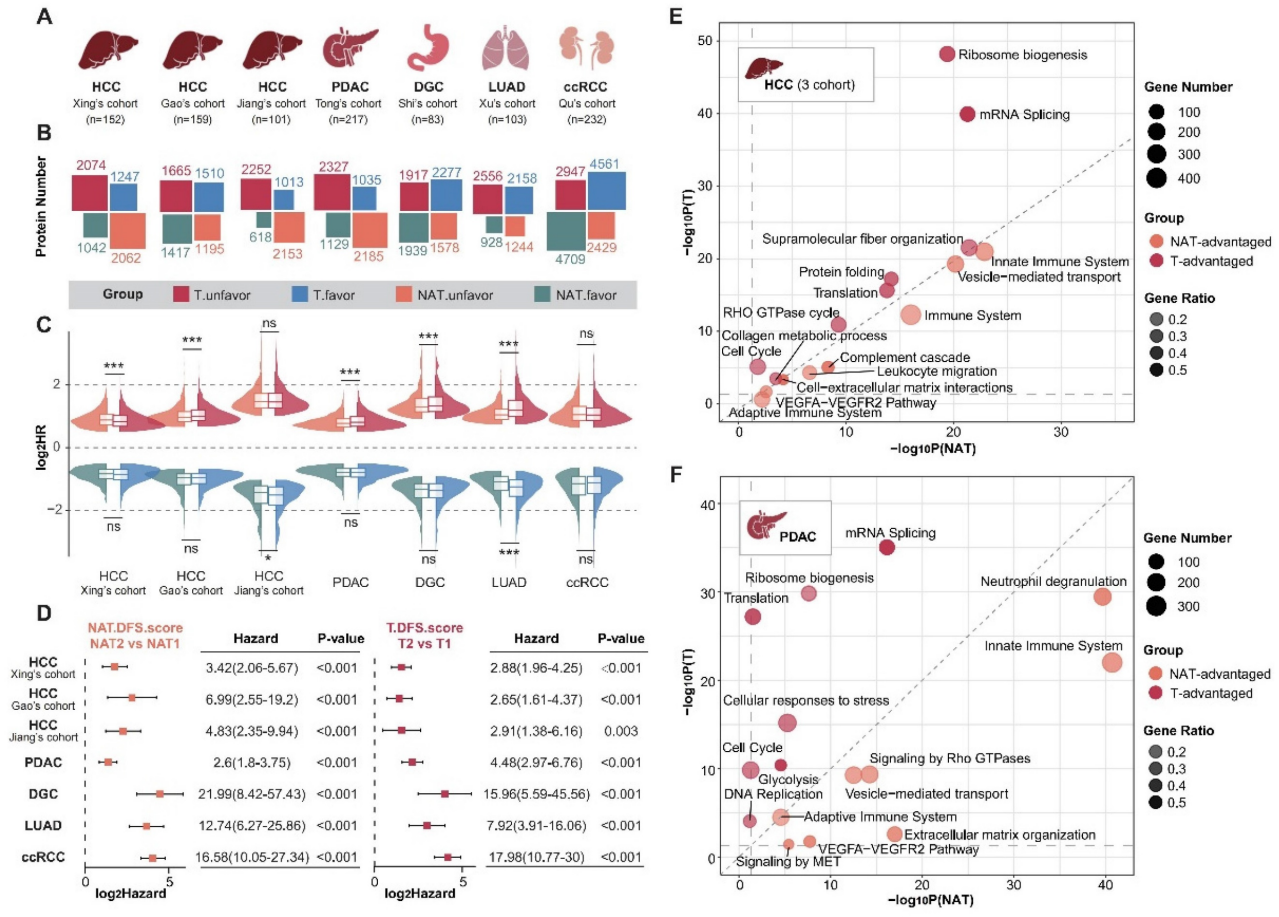
** NATs exhibited significantly different prognostic-related functions and comparable prognostic predictive performance compared with tumor tissue.** (A) Introduction to analyzing data. (B) Overview of the number of recurrence-related proteins in tumors and NATs. (C) HR distribution of recurrence-related proteins in tumors and NATs. (D) Recurrence-subgroups based on tumors or NATs can reliably predict recurrence. (E and F). Functional enrichment analysis for unfavorable proteins of tumors and NATs in HCC (E) and PDAC (F). Statistical test: Wilcoxon. *P<0.05, **P<0.01, ***P<0.001. HCC: hepatocellular carcinoma; PDAC: pancreatic ductal adenocarcinoma; DGC: diffuse gastric cancer; LUAD: lung adenocarcinoma; ccRCC: clear cell renal cell carcinoma.

To systematically analyze the roles of tumors and NATs in the process of cancer recurrence, we collected proteomic data and corresponding clinical information for seven sets of solid tumors 13-15, 27-30, including hepatocellular carcinoma (HCC), pancreatic ductal adenocarcinoma (PDAC), diffuse gastric cancer (DGC), lung adenocarcinoma (LUAD), and clear cell renal cell carcinoma (ccRCC) (Figure 1A; Supplementary Table S1 and S2). The hazard ratios (HR) of recurrence were calculated using log-rank tests based on a Cox proportional hazards regression model. Proteins with a P-value of less than 0.05 were defined as recurrence-related proteins. HR higher than 1 was defined as unfavorable, while HR less than 1 was described as favorable. Analysis revealed that the recurrence prediction proteins in the NATs were comparable to those in the tumor tissues in terms of both the number and distribution of HRs (Figure 1B and C).

Distinct sets of prognosis-related proteins were identified in both tumors and NATs, and the intersection of three sets of HCC data was selected for further analysis. DFS scores were obtained through ssGSEA and defined as ES_unfavor_ minus ES_favor_. We divided the subtypes according to the best cut-off of the tumor and NAT DFS scores respectively. Results showed that tumors and NATs could predict recurrence stably and patients could be divided into two groups with significant recurrence differences (Figure 1D; Supplementary Figure S1A). The group with a poor prognosis is defined as subtype T2 or NAT2. The recurrence rate is significantly higher in patients with subtype T2 and NAT2 compared to those with subtype T1 and NAT1 in all cohorts, with a difference of over 30% (HR > 2). In addition, in HCC, LUAD, and DGC cohorts, the HR between subgroups corresponding to NAT seemed to be higher than that of tumor subgroups (Supplementary Figure S1B).

Functional enrichment analysis was conducted to compare the similarities and differences in function between tumors and NATs by examining the unfavorable and favorable proteins. Taking HCC and PDAC as examples, it was found that prognostic-related proteins in tumor tissues are mainly enriched in ribosome biosynthesis, alternative splicing, translation, the cell cycle, and other pathways. These findings correspond to the characteristics of tumor escape from growth inhibition and continuous proliferation. In contrast, prognostic proteins in NATs exhibit more prominent functions related to innate immunity, complement activation, extracellular matrix, vesicle transport, and angiogenesis. This suggests that changes in these functional modules of NATs provide a suitable environment for tumor recurrence (Figure 1E and F; Supplementary Table S3).

### The NATs complement tumor clinical and molecular features and are irreplaceable

**Figure 2:**
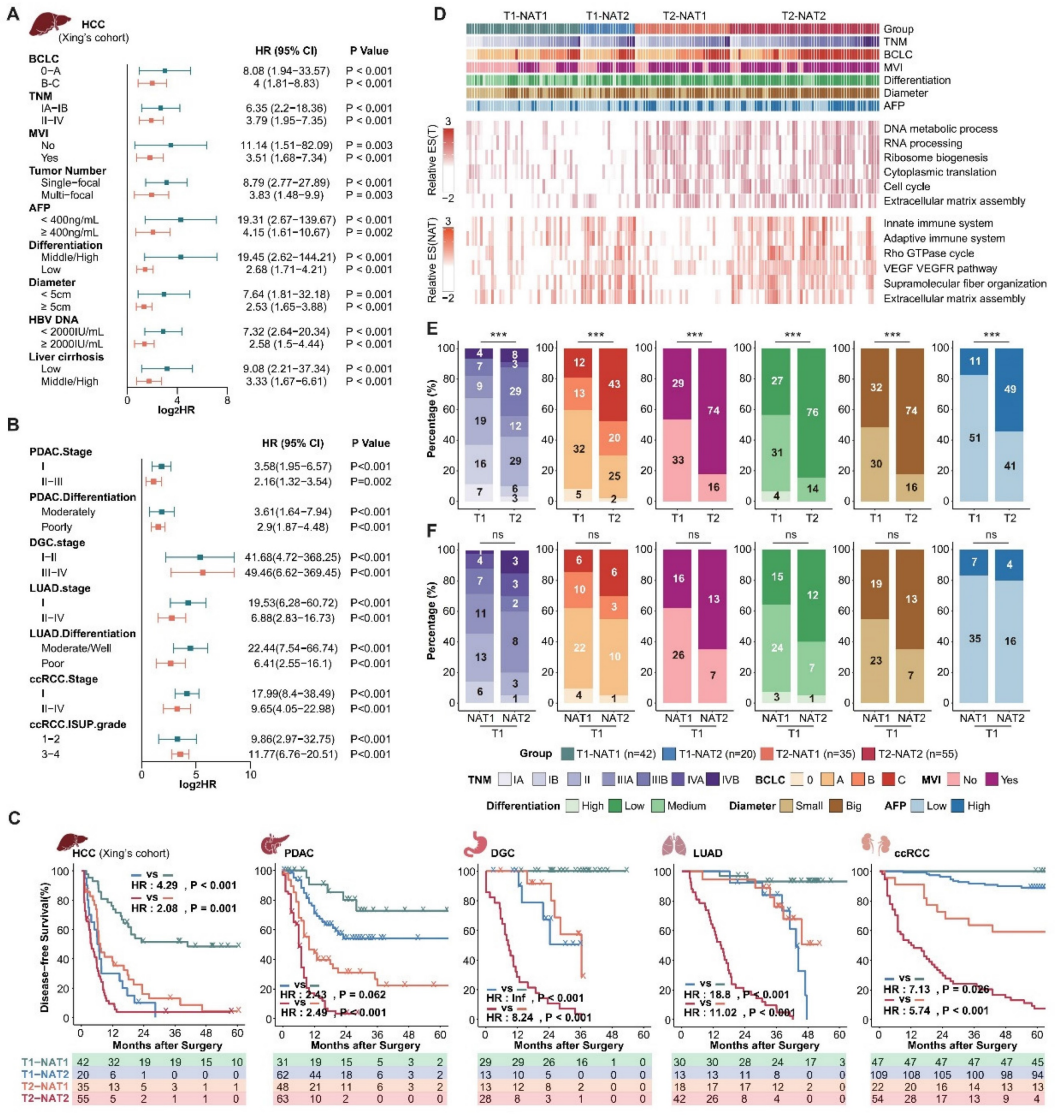
** NAT subgroups complement clinical and tumor molecular features and are irreplaceable.** (A and B), NAT subgroups can stably stratify the recurrence risk for patients with high and low recurrence risk defined by clinical indicators and staging. (C) Integrating tumor and NAT recurrence subgroups can achieve more accurate recurrence risk assessment. (D) Overview of clinical and molecular characteristics of differentially recurrence subgroups. (E) Comparison of clinical characteristics among tumor recurrence subgroups. (F) Compared with the T1-NAT1 group, the T1-NAT2 group did not show significantly more aggressive clinical features. Statistical test: Chi-Squared Test.

Next, we aimed to investigate whether NATs could complement tumor clinical and molecular features. Given the comprehensive clinical information available from Xing's cohort (HCC), we choose to use this cohort as a representative example to illustrate the relationship between DFS score, tumor score, and clinical indicators. Incorporating DFS scores of tumors and NATs, MVI, tumor diameter, AFP, differentiation, and Serum HBV-DNA, we further verified that the DFS score of the NATs is an independent prognostic risk factor relative to the tumor and clinical features through multiple Cox regression analysis (N.DFS.socre-Xing's cohort: multivariate hazard ratio = 2.389; P-value < 0.001; Supplementary Table S4). Additionally, the NAT DFS score can further effectively stratify subgroups with different recurrence risks determined by clinical tumor grade and various indicators (Figure 2A). Expanded to other datasets, the NAT subgroups can stably stratify the recurrence risk for patients with high and low recurrence risk defined by clinical indicators and staging, confirming that the NAT features can complement clinical indicators (Figure 2B; Supplementary Figure S2A and B). Furthermore, the DFS score of NATs can also complement tumor recurrence subgroups. Based on the tumor DFS score, the cohort was divided into T1 and T2 groups with different recurrence rates. NAT DFS score was further used to divide T1 and T2 into two groups (T1-NAT1, T1-NAT2; T2-NAT1, T2-NAT2). The recurrence rate of different NAT subgroups in the same T group was still significantly different. Generally speaking, subtype T2-NAT2 had the worst prognosis, subtype T1-NAT1 had the best prognosis, and the other two subtypes had a moderate prognosis (Figure 2C; Supplementary Table S5).

Taking Xing's cohort as an example, we compared the molecular and clinical features of the four subgroups of tumors and NATs (Figure 2D). Compared to the T1 group, the tumor-related pathways enrichment score of the T2 group was significantly higher, corresponding to more active proliferation-related pathways such as the cell cycle and alternative splicing. Consistently, the T2 group exhibited a more robust invasive phenotype in clinical manifestation, corresponding to a significantly higher proportion of an advanced TNM stage, MVI, poorly differentiated tumors, large tumors (Diameter > 5 cm), and abnormal AFP elevation (Figure 2D and E). It is important to note that, compared to the T1-NAT1 group, the tumor molecular features of the T1-NAT2 group did not show significant differences (Figure 2D). Additionally, although their clinical characteristics have different trends, there are no significant differences between the two groups (Figure 2F). The only abnormal activity was observed in pathways such as immunity, extracellular matrix, and angiogenesis in the NAT, and the enrichment scores of these characteristic pathways in NATs were significantly higher than those of the tumor tissues in the T1-NAT2 subgroup of 3 LIHC cohorts (Figure 2D, Supplement Figure S3). This indicates that for tumor prognosis assessment and anti-recurrence therapy, in-depth studies targeting tumor molecular features and clinical indicators cannot replace the corresponding exploration of NAT. Thus, it is important to pay attention to in-depth analysis and intervention of the microenvironment of the NATs for T1-NAT2 patients, which is different from the traditional tumor-dominated prevention and treatment strategies.

### The extracellular matrix is correlated with postoperative recurrence in both tumors and NATs, and there is complementarity between the two

**Figure 3:**
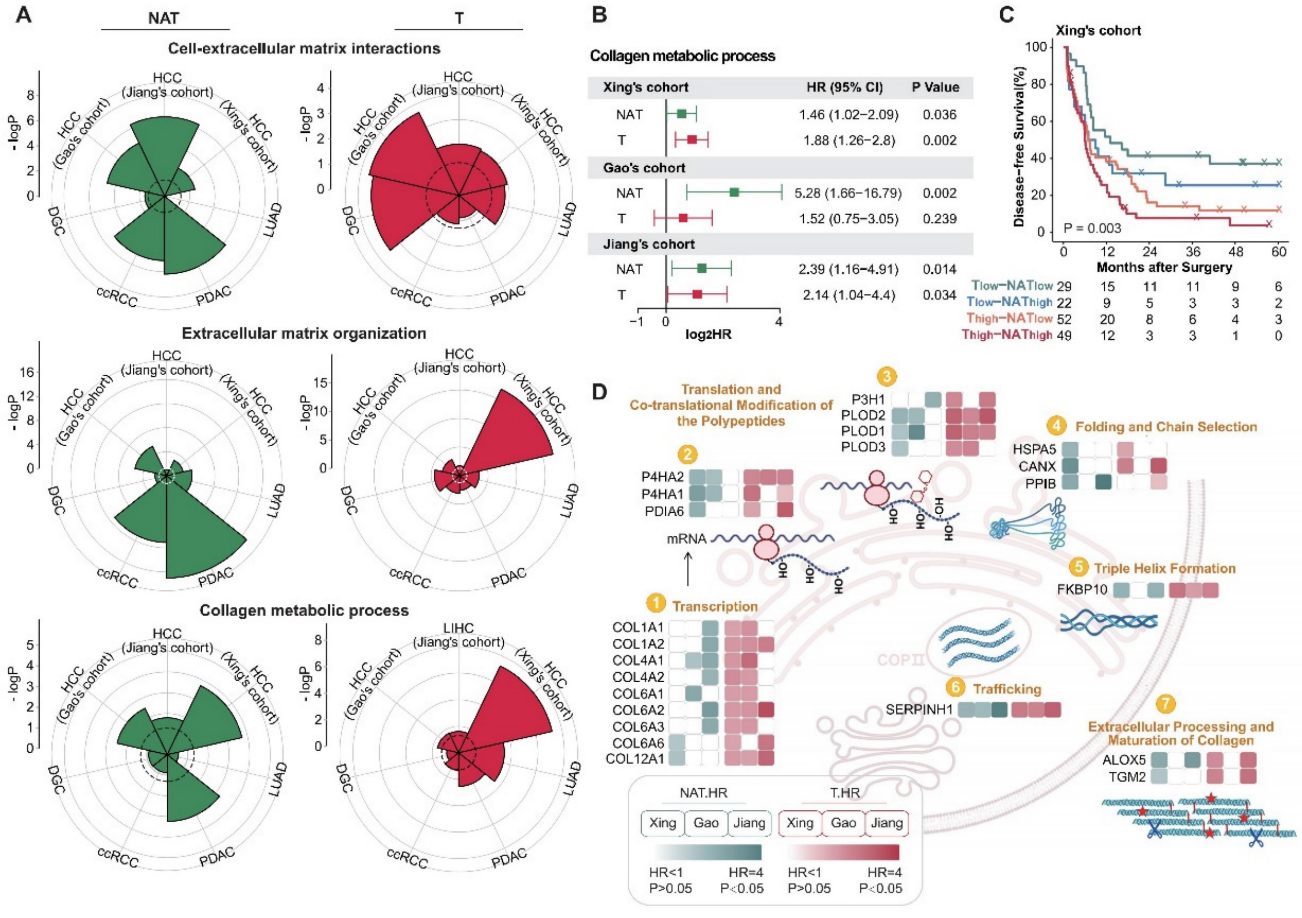
** The extracellular matrix is correlated with postoperative recurrence in both tumors and NATs.** (A) Recurrence-related proteins in tumors and NATs of multiple cancer types are enriched in extracellular matrix-related pathways. The dotted circle corresponds to a P-value threshold of 0.05. (B) Active collagen metabolic represents a high risk of postoperative recurrence in HCC cohorts, both in tumors and NATs. (C) Patients can be divided into 4 subgroups with significant DFS differences based on the collagen metabolism pathway of tumors and NATs. (D) Overview of proteins involved in the collagen biosynthesis pathway. Most proteins in the pathway show unfavorable trends in both tumors and NATs. HCC: hepatocellular carcinoma; PDAC: pancreatic ductal adenocarcinoma; DGC: diffuse gastric cancer; LUAD: lung adenocarcinoma; ccRCC: clear cell renal cell carcinoma.

To carry out efficient and precise interventions for postoperative patients, we place special emphasis on functional modules with prognostic significance in both tumor and NAT. Further functional enrichment analysis reveals that, in addition to proliferation and immune-related functions, extracellular matrix-related pathways play a crucial role in both tumors and NATs (Figure 1E and F, Figure 2D). Studies have shown that the extracellular matrix and collagen pathways participate in and promote fibrosis, tumorigenesis, invasion, and drug resistance 31-34. More detailed functional module analysis revealed that in both tumors and NATs, extracellular matrix subclasses such as "Cell-extracellular matrix interactions", "Extracellular matrix organization," and "Collagen metabolic process" are active, suggesting a high risk of recurrence, especially in HCC and PDAC (Figure 3A). To more comprehensively characterize the profile of ECM and collagen metabolism in HCC with different prognosis, and then find relevant recurrence risk markers and intervention targets, we conducted an integrated analysis of three publicly available HCC proteomic data sets.

The activity levels of collagen metabolism pathways in the tumors and NATs of HCC were evaluated using ssGSEA methods. It was found that collagen metabolism pathways were indicative of poor prognosis in both the tumor and NAT of HCC, and there was also a certain degree of complementarity (Figure 3B and C). We then focused on the predictive performance of key proteins in collagen synthesis metabolism pathways in the tumors and NATs of HCC. Most of these proteins belonged to unfavorable proteins in both the tumors and NATs, further corroborating the role of collagen metabolism in promoting HCC recurrence. Among them, SERPINH1 (HSP47), as a molecular chaperone involved in collagen folding 35, is the only one that could stably identify high-risk recurrent patients in both tumors and NATs of all HCC cohorts and has corresponding targeted drugs, making it a potential high-performance recurrence prediction marker and drug target (Figure 3D).

### SERPINH1 is a potential marker with predictive capability for recurrence in both tumors and NAT

**Figure 4:**
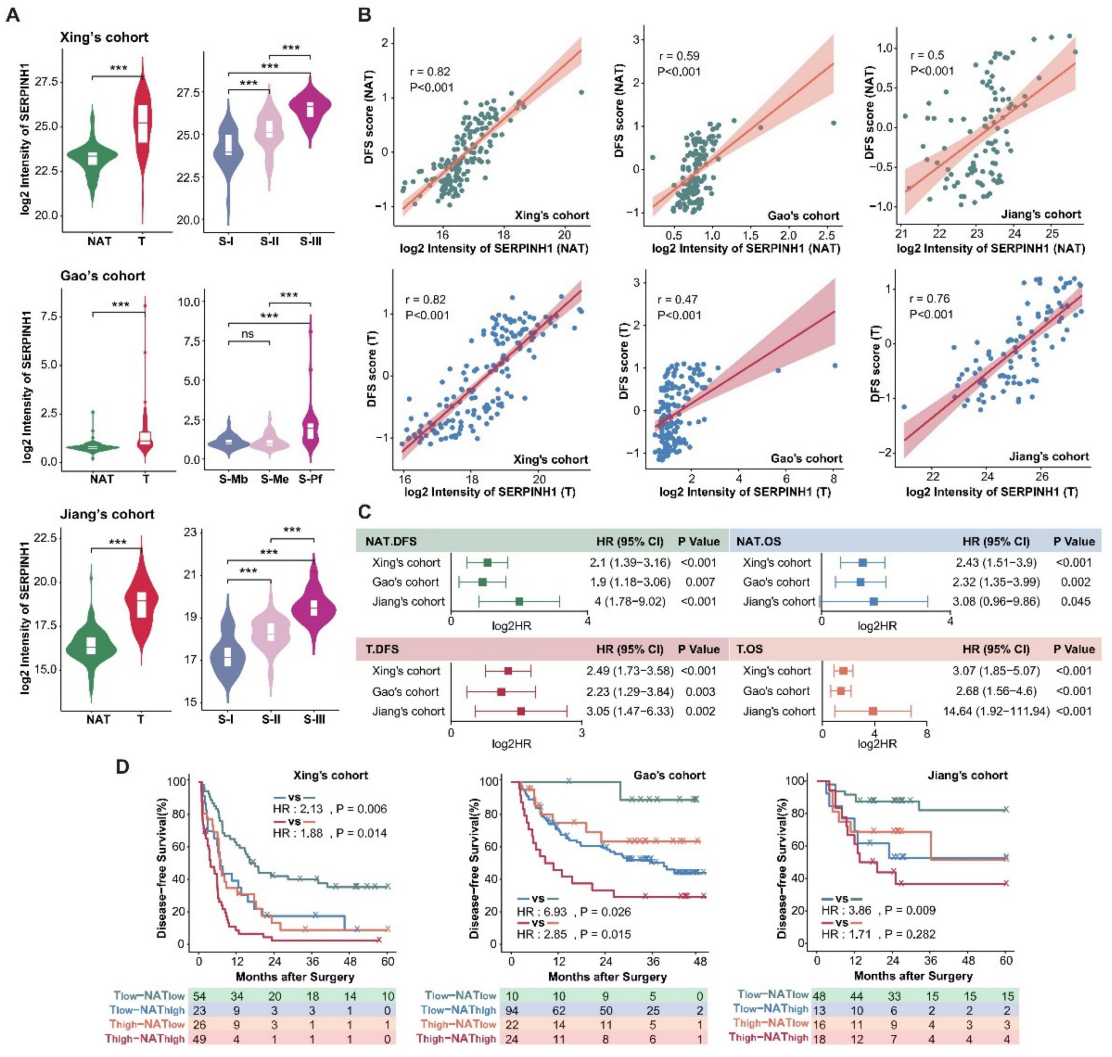
** SERPINH1 is a potential marker with predictive capability for recurrence in both tumors and NATs.** (A) Compared to the NATs, SERPINH1 was significantly upregulated in HCC tumors, especially in the most malignant tumor subtypes with the worst prognosis. (B) The expression levels of SERPINH1 showed significant positive correlations with DFS scores in tumors and NATs, respectively. (C) The high expression of SERPINH1 in the tumors and NATs had stable unfavorable effects on both disease-free survival and overall survival. (D) Based on the expression level of SERPINH1 in the tumors and NATs, patients could be divided into four groups with different recurrence rates, similar to DFS scores.

As a protein with the most stable ability to stratify the risk of recurrence in both tumors and NATs of three HCC cohorts (Figure 3D), SERPINH1 exhibits the following characteristics: Firstly, compared to the NATs, SERPINH1 was significantly upregulated in tumors, and its expression increased in a stepwise manner with increasing malignancy in three HCC tumor subtypes (Figure 4A). Secondly, the expression level of SERPINH1 was both positively correlated with the DFS score, suggesting it could represent the recurrence-related molecular characteristics of both the tumors and NATs (Figure 4B; Supplementary Figure S4A). Finally, the expression level of SERPINH1 in the tumors and NATs also had stable predictive effects on overall survival (OS) (Figure 4C). Based on the expression level of SERPINH1 in the tumors and NATs, patients could also be divided into four subgroups with different recurrence rates. Patients with low expression of SERPINH1 in both tumors and NATs have the best prognosis, while patients with high expression of SERPINH1 have the highest recurrence rate, resulting in a 40% difference between the two groups. (Figure 4D).

**Figure 5:**
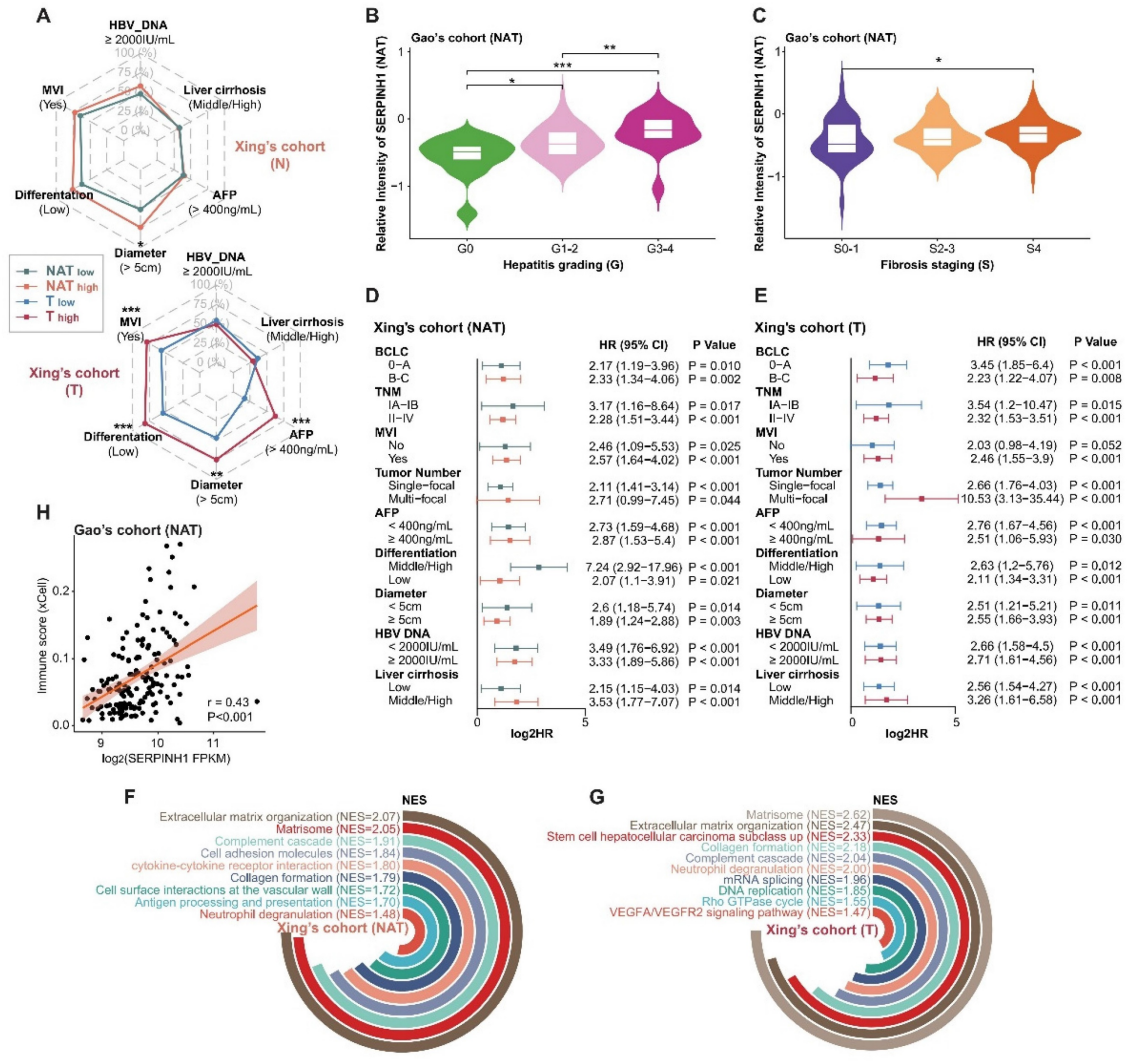
** Clinical and molecular features of the high SERPINH1 expression groups in tumors and NATs.** (A) High expression of SERPINH1 in tumors was accompanied by abnormal elevation of serum AFP. (B and C) According to the pathological scores of inflammation and fibrosis, SERPINH1 was significantly upregulated in the severe inflammation (B) and fibrosis groups (C). The G score (0-4) represents the grading system for inflammation while the S score (0-4) represents the staging system for fibrosis to evaluate the level of chronic hepatitis. The score is positively correlated with the severity. (D and E) The expression of SERPINH1 in tumors (E) and NATs (D) can stably stratify the recurrence risk for patients with high and low recurrence risk defined by clinical indicators and staging. (F and G) The circle track plot shows the functional pathways that are significantly enriched in the SERPINH1 high-expression group compared with the low-expression group in tumor (G) and NATs (F). (H) The expression of SERPINH1 showed significant positive correlations with immune scores in NATs. NES, normalized enrichment score. Statistical test: Chi-square test, * <0.05, **P <0.01, ***P <0.001.

Analysis of the clinical features of the high SERPINH1 expression group revealed that this group in tumors was associated with a higher proportion of abnormally elevated serum AFP (Figure 5A). Transcriptional data from human fetal liver development indicated SERPINH1 expression was significantly higher in fetal liver than in postnatal liver tissues 36. Single-cell data on human fetal liver development suggested that SERPINH1 is mainly localized in vascular endothelial cells and fibroblasts, and the proportions of these two cell types decrease during liver development (Supplementary Figure S4B and C) 37. Similarly, single-cell data on liver cancer also suggested that SERPINH1 is mainly localized in vascular endothelial cells and fibroblasts (Supplementary Figure S4D) 38. The Gao's cohort provided detailed pathological scores for inflammation and fibrosis, showing significant upregulation of SERPINH1 in the NATs of severe inflammation and fibrosis groups (Figure 5B and C). Importantly, SERPINH1 was able to identify high-risk recurrence patients even among those clinically identified as low-risk, indicating that SERPINH1 expression levels complement existing clinical features for risk assessment (Figure 5D and E).

We further analyzed the molecular features of the high SERPINH1 expression group. We found that tumors and NATs in the high SERPINH1 expression group were significantly enriched in the extracellular matrix and collagen formation pathways (Figure 5F and G; Supplementary Table S6). Additionally, consistent with the pathological inflammation and fibrosis score, the expression level of SERPINH1 in the NATs was positively correlated with immune scores, accompanying abnormal activity in the complement system and neutrophils, indicating an inflammatory immune environment in the NATs with high SERPINH1 expression (Figure 5F and H). Consistent with the high AFP abnormality in the high SERPINH1 expression group in tumors, the high SERPINH1 expression group in tumors also showed enrichment of tumor stemness pathways, along with abnormal activity in unfavorable pathways such as alternative splicing and DNA replication (Figure 5G).

In summary, SERPINH1 can represent unfavorable molecular features in both tumors and NATs, and the integration of SERPINH1 expression in tumors and NATs can effectively assess the risk of recurrence after liver resection.

### SERPINH1 exhibits stable predictive efficacy for recurrence in general and low-recurrence risk population validation cohorts

**Figure 6:**
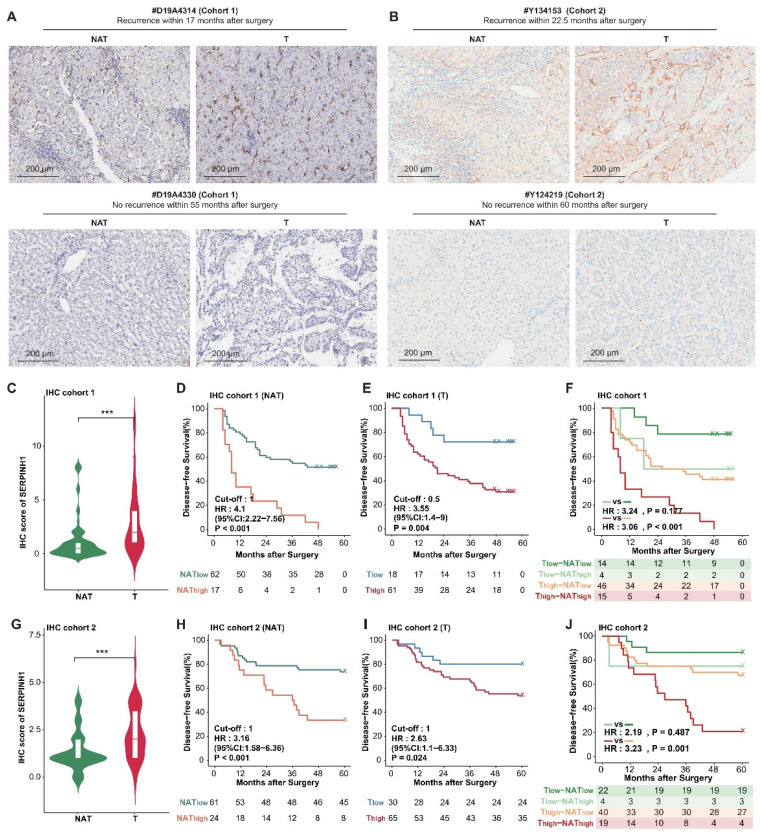
** SERPINH1 exhibits stable predictive efficacy for recurrence in both the general population and low-recurrence risk population validation cohorts.** (A and B) Examples of immunohistochemistry with strong staining and weak staining in cohort 1 (A) and cohort 2 (B). (C and G) IHC scores of SERPINH1 in tumor tissues were significantly higher than those in NATs. (D, E, H, and I) SERPINH1 expression in tumors and NATs can exert stable predictive efficacy for recurrence in both the general population and clinically low-risk population. (F and J) Based on the IHC score of SERPINH1 in the tumors and NATs, patients could be divided into four groups with different recurrence rates. Cohort 1(general population): 79 patients, 50 cases of TNM stage T1, 26 cases of T2, and 3 cases of T3. Cohort 2(clinically low-recurrence risk population): 79 patients, all patients were TNM stage T1, had no MVI, and tumor diameter was less than or equal to 5 cm.

To further validate the predictive efficacy of SERPINH1 for recurrence, we enrolled two independent validation cohorts consisting of tumor and paired NAT samples and validated them using immunohistochemistry (IHC). The IHC staining of SERPINH1 is mainly localized in the tumor stroma, with clear distinctions in staining intensity. The representative staining is shown in Figure 6A and B.

Cohort 1 comprised 85 cases of pathologically confirmed primary hepatocellular carcinoma, including 79 cases with paired NAT samples, with postoperative follow-up information exceeding 3 years (Supplementary Table S7). Only paired samples were included in the analysis. Among them, there were 50 cases of TNM stage T1, 26 cases of T2, and 3 cases of T3; 21 cases had vascular invasion, 50 cases did not; 33 cases had large tumors (diameter greater than or equal to 5 cm), and 46 cases had small tumors; 29 cases had abnormal elevation of serum AFP (greater than or equal to 400 ng/mL), and 50 cases were relatively normal, with a 5-year recurrence rate of 59.5%. IHC scores from tumors were significantly higher than those of NATs (T-average: 2.86, T-max: 12; N-average: 1.19, N-max: 8; P<0.001; Figure 6C). The intensity of SERPINH1 expression in both tumors and NATs independently stratified patients into different recurrence risk groups (hazard ratio 3.55, 1.4 to 9; P-value = 0.004; hazard ratio 4.1, 2.22 to 7.56; P-value < 0.001 ) (Figure 6D and E). Integrating the expression of SERPINH1 in tumors and NATs can divide patients into four groups with different prognoses. Among them, the subgroup with high expression of SERPINH1 in both tumor and NAT corresponds to patients who all relapsed within 5 years (Figure 6A and F).

Cohort 2 included a total of 95 samples of primary hepatocellular carcinoma, with 85 cases having corresponding NATs and postoperative follow-up information for up to 5 years (Supplementary Table S8). All patients were TNM stage T1, had no MVI, and tumor diameter was less than or equal to 5 cm, indicating a clinically low recurrence risk population 16. Among them, 18 cases had serum AFP greater than 400ng/mL (21.2%), and the 5-year recurrence rate was 37.6%. Compared to the NATs, the IHC score of SERPINH1 was significantly higher in the tumor tissues (T-average: 2.29; N-average: 1.38; P<0.001; Figure 6G). The level of SERPINH1 expression in both tumors and NATs also demonstrated a strong predictive capability for recurrence risk (hazard ratio 2.63, 1.1 to 6.33; P-value = 0.024; hazard ratio 3.16, 1.58 to 6.36; P-value < 0.001) (Figure 6H and I), as observed in cohort 1. Similarly, the prognostic stratification was conducted through the integration of tumor and NAT expression levels. Patients with elevated SERPINH1 in both tumor and NATs (T2-NAT2) exhibited the highest risk of recurrence, with a 5-year DFS rate reaching 78.95% (Figure 6B and J).

In conclusion, we have confirmed in 2 independent immunohistochemistry cohorts that SERPINH1 serves as a reliable biomarker applicable to both the general population and clinical low-risk groups.

### Targeting SERPINH1 can delay the occurrence and development of HCC

**Figure 7:**
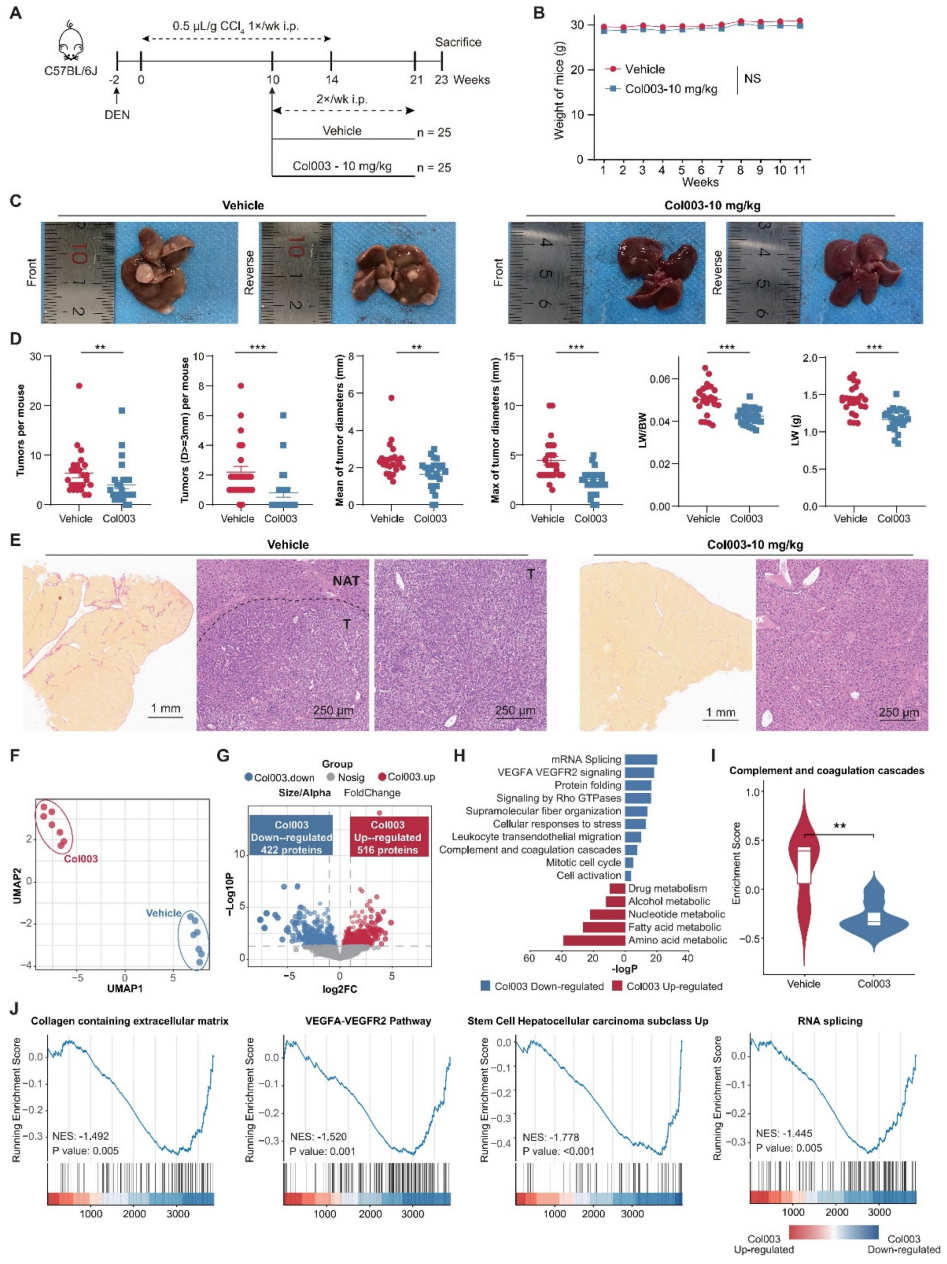
** Inhibition of SERPINH1 activity by Col003 suppresses the progression of murine hepatocellular carcinoma progression.** (A) Schematic Illustration of the DEN/CCl_4_-induced liver cancer and Col003 treatment strategy in mice. (B) Body weight curves of mice in control (vehicle-treated) and treatment (Col003-administered) groups. (C) Representative gross tumor images from both control (vehicle-treated) and treatment (Col003-administered) groups of mice. (D) The impact of Col003 intervention on liver tumor burden, including the count of all tumors and those ≥3mm in diameter, alongside metrics of tumor size, maximal tumor dimension, liver-to-body weight ratio, and murine body weight. (E) Representative images of mouse liver tissue sections stained with H&E (Left) (Scar bar, 1 mm) and Sirius Red (Right) (Scale bar, 250 μm) from the control and treatment groups. (F) UMAP analysis showed significant clustering differences between the Col003-treated group and the control group. (G) The volcano plot showed significantly changing genes in the Col003-treated group. (H) Col003 down-regulated genes were mainly enriched in the unfavorable pathways of HCC tumors and NATs. (I) Box plots indicated that the complement and coagulation cascade pathway in the Col003-treated group was significantly down-regulated. (J) GSEA analysis of representative unfavorable pathways in tumors and NATs. The above pathways were significantly down-regulated in the Col003-treated group. Data from (B and D) was presented as Mean SEM, n=25 for each group. (B) was analyzed using two-way analysis of variance (ANOVA), while (D) was subjected to the two-tailed Mann-Whitney U test. NS >0.05, **P <0.01, ***P <0.001. D, diameter; LW, liver weight; BW, body weight. UMAP, Uniform Manifold Approximation and Projection; NES, normalized enrichment score.

Subsequently, we aimed to ascertain whether SERPINH1 is a potential target for HCC therapy. Leveraging the well-documented role of SERPINH1 in fibrosis across various contexts 39, we employed the DEN-CCl_4_-induced tumor model in mice (Figure 7A), which accurately simulates HCC progression in the context of hepatic fibrosis 40, 41. By ten weeks following the initiation of CCl_4_ administration via intraperitoneal injection, both reported data 41 and our representative data of gross images and Sirius Red staining results (Supplementary Figure S5) confirmed the establishment of hepatic fibrosis in the mice.

Subsequently, Col003, a specific SERPINH1 inhibitor known to disrupt its interaction with collagen and thus inhibit collagen secretion by destabilizing the collagen triple helix 42, was administered at 10 mg/kg twice weekly for 11 weeks. Two weeks after the final administration, mice were euthanized for gross and immunohistochemical analysis of liver tissues. Our findings revealed that Col003 did not markedly affect murine body weight, implying a favorable safety profile for the 10 mg/kg Col003 (Figure 7B). Importantly, Col003 treatment significantly alleviated the tumor burden in the liver of these mice, demonstrated by a decrease in both tumor number and diameter, as well as a reduction in liver weight and liver weight to body weight ratio when compared to the vehicle group (Figure 7C and D; Supplementary Table S9). Also, H&E staining verified the occurrence of HCC in the control group, whereas the liver tissue in the Col003-treated group exhibited smaller or even absence of tumors in the cross-section. Furthermore, Sirius Red staining revealed that the Col003 treatment group exhibited a reduction in liver fibrosis compared to the control group (Figure 7E). These results underscore SERPINH1 activity inhibition as an effective strategy to constrain HCC development, positioning SERPINH1 as a promising target for pharmacological intervention in HCC.

### Targeting SERPINH1 can downregulate functional pathways such as extracellular matrix, tumor stemness, alternative splicing, and complement activation

To elucidate the impact of inhibiting SERPINH1 activity on the liver molecular phenotype in the DEN-CCL_4_-induced mouse model, we compared the proteomic data of 14 liver tissues from the Col003-treated group and the control group, respectively (Supplementary Table S10). UMAP analysis demonstrates a clear distinction between the proteomes of the two groups, indicating that Col003 treatment had a greater impact on the molecular characteristics of the liver (Figure 7F) In the Col003-treated group, 422 proteins were significantly downregulated (FC<1, P<0.05), while 516 proteins were significantly upregulated (FC>1, P<0.05). Notably, approximately 91.5% of the downregulated proteins in the treated group were associated with unfavorable outcomes in the tumors or NATs (Figure 7G; Supplementary Figure S6; Supplementary Table S11). Functional enrichment analysis showed that pathways such as angiogenesis (VEGFA-VEGFR2) and complement cascade were significantly downregulated in the NATs, while alternative splicing and cell cycle pathways were suppressed in the tumor tissues after Col003 treatment. Conversely, the upregulated proteins in the Col003-treated group enriched in liver metabolic pathways such as drug metabolism, alcohol metabolism, and fatty acid metabolism, indicating that the liver function of the Col003-treated group was closer to that of normal liver tissues (Figure 7H and I; Supplementary Table S12). Furthermore, GSEA analysis revealed significant downregulation of representative pathways associated with high SERPINH1 expression—such as collagen metabolism, angiogenesis, tumor stemness, and alternative splicing—in the Col003-treated group (Figure 7J).

To conclude, the proteomic analysis further confirmed that Col003 can significantly downregulate unfavorable functional pathways related to recurrence, effectively reverse recurrence-related molecular characteristics of the liver, and thereby delay the occurrence and progression of liver cancer.

## Discussion

Early detection of high-risk recurrence and effective strategies to prevent recurrence after liver cancer surgery are currently challenging issues in the field 3. Unlike patients with advanced-stage HCC who have long-term tumor burden, postoperative HCC patients lack targetable tumor tissue over a long period. Therefore, the existing management strategies that solely focus on targeting tumor tissue are inadequate. This may explain why TACE and sorafenib did not improve the prognosis of patients as postoperative adjuvant treatments 17, 43. According to the "seed and soil" theory, both the malignant phenotype of the tumor cells and the corresponding microenvironment contribute to recurrence 18, 19. Consistent with the theory, studies have shown that NATs can reliably predict late recurrence 20, 21, and interventions targeting NATs can significantly prolong recurrence-free survival following surgery 25. Therefore, NAT has garnered growing attention as the "soil" for tumor development.

Currently, there is limited research on NATs, with previous studies mainly concentrated in the field of liver cancer. Liao *et al.* highlighted the heterogeneity of metabolism and the microenvironment in the NATs, suggesting that proteomic analysis of NATs can stratify patients into three subgroups with differential prognoses 44. Gu *et al.* further introduced the peritumor microenvironment (PME), which includes tissue surrounding the tumor that is very close to the “soil”, to describe the mechanisms underlying the occurrence and progression of HCC. They indicated that PME differs from normal tissues and plays different roles in tumor occurrence and progression stages and identified three PME subtypes with distinct molecular and immune features, among which the S-III subtype has a shorter overall survival time. TYMP protein was identified as a characteristic S-III subtype protein and validated as an anti-angiogenic target in an orthotopic mouse model of hepatocellular carcinoma 22. Zhu *et al.* provided a detailed description of the heterogeneity of NATs and the differences between NATs and healthy livers and revealed that molecular features of tumor subgroups in HCC were partially reflected in their respective NATs.

They identified two NAT subgroups and characterized them in detail. Subtype II has a worse prognosis, with NATs resembling tumor tissues, showing more active extracellular matrix and adhesion-related pathways 45. Gong *et al.* performed subtype and prognosis prediction based on the enrichment scores of immunologic and hallmark gene sets in tumors and NATs, and used LASSO regression to obtain 3 tumor gene sets and 4 NAT gene sets that can predict postoperative recurrence, suggesting both tumor and NAT play an important role in the recurrence process 46. These studies confirmed that NATs in HCC differ from normal liver tissues, exhibit heterogeneity, and can affect tumor biology and patient outcomes. However, most studies have been conducted on single-center cohorts and are limited to describing the heterogeneity of adjacent tumors and their correlation with clinical characteristics. Feasible management strategies for NATs are still lacking, and it remains unclear whether the biological characteristics and impact on clinical outcomes of NATs are specific to liver cancer or have pan-cancer universality. Considering the reliability of the research, conclusions from multicenter cohorts are more robust and reproducible 47. Therefore, this study integrated seven proteomic datasets to systematically analyze the predictive efficacy and functional emphasis of tumors and NATs in five tumors, demonstrating the pan-cancer predictive ability of NATs. Patients with unfavorable phenotypes in the NAT should be identified and managed differently from traditional anti-tumor therapies. Based on three HCC proteomic datasets, this study explores the feasibility of integrating tumors and NATs for biomarker and drug target development, providing new insights for tumor recurrence research and postoperative management. SERPINH1 has been identified as a promising biomarker and drug target for predicting postoperative recurrence in both the tumor and its NAT.

SERPINH1, also known as HSP47, belongs to the heat shock protein family, is involved in collagen synthesis and quality control, and plays a role in atherosclerosis and various fibrotic diseases 35, 48. Knockout of *Serpinh1* in mice leads to death due to the basement membrane disruption caused by abnormal synthesis of type IV collagen 49. Heat shock proteins are considered potential activators of innate immunity that induce monocyte-macrophage systems to produce pro-inflammatory cytokines and are involved in antigen presentation and tumor immunity 50. Recent studies have shown that SERPINH1 can enhance the interaction between cancer cells and platelets, promoting tumor metastasis 51, while downregulation of SERPINH1 can attenuate immune cell activation and form neutrophil extracellular traps 52. Data analysis derived from the clinical LIHC cohorts and mice treated with SERPINH1 inhibitors further substantiated the reproducibility of these phenomena. As shown in Supplementary Table S13, higher abundance of SERPINH1 in tumor tissues is associated with increased activity in pathways related to platelet, neutrophil, and epithelial-mesenchymal transition. In NAT tissues, higher abundance of SERPINH1 is also linked to increased activity in platelet and neutrophil-related pathways, although the epithelial-mesenchymal transformation did not show significant changes. Similarly, mouse models treated with Col003 exhibited down-regulation of pathways associated with platelet, neutrophil, and epithelial-mesenchymal transformation (Supplementary Figure S7). In terms of prognosis, SERPINH1 is unfavorable in most tumors and NATs analyzed in this study (Figure 4C, Figure S8). Pan-cancer analysis based on TCGA data also indicates abnormal expression of SERPINH1 in various tumors, with its high expression correlated with lower overall survival rates of patients in 11 types of tumors 53. In addition, this study has informed that BMS-986263, an inhibitor targeting SERPINH1, may inhibit the occurrence and development of liver cancer. BMS-986263 is a lipid nanoparticle delivering small interfering RNA targeting SERPINH1 mRNA, and it has shown promising safety and significant improvement in fibrosis status in a phase II trial for the treatment of advanced fibrosis in hepatitis C patients 54. This study also verified that targeting SERPINH1 can effectively reduce liver fibrosis, but its effect on hepatitis is not obvious (Supplementary Figure S9). This study expands its potential in the treatment of liver cancer and is expected to broaden its new indications. However, it is important to note that there is currently a lack of recognized animal models simulating tumor recurrence and HBV infection in HCC. Considering the similarity between postoperative recurrence of HCC and spontaneous tumorigenesis and the ability of fibrosis-to-tumor mouse models to simulate the cirrhotic background of HCC patients, we selected the DEN-CCI4 murine model as the validation model. However, it may not fully reflect real-world application scenarios, and further investigation on more appropriate experimental models is required. Due to the spontaneous and diffuse tumorigenesis of the DEN-CCI4 murine model, our sampling was limited and we were unable to accurately distinguish between tumor and non-tumor tissues, resulting in the inability to separately describe the effects of targeting SERPINH1 on tumor and NAT tissues. More ideal animal models and complete experimental designs are needed to further confirm the conclusions from the proteomic data.

## Conclusions

**Figure 8:**
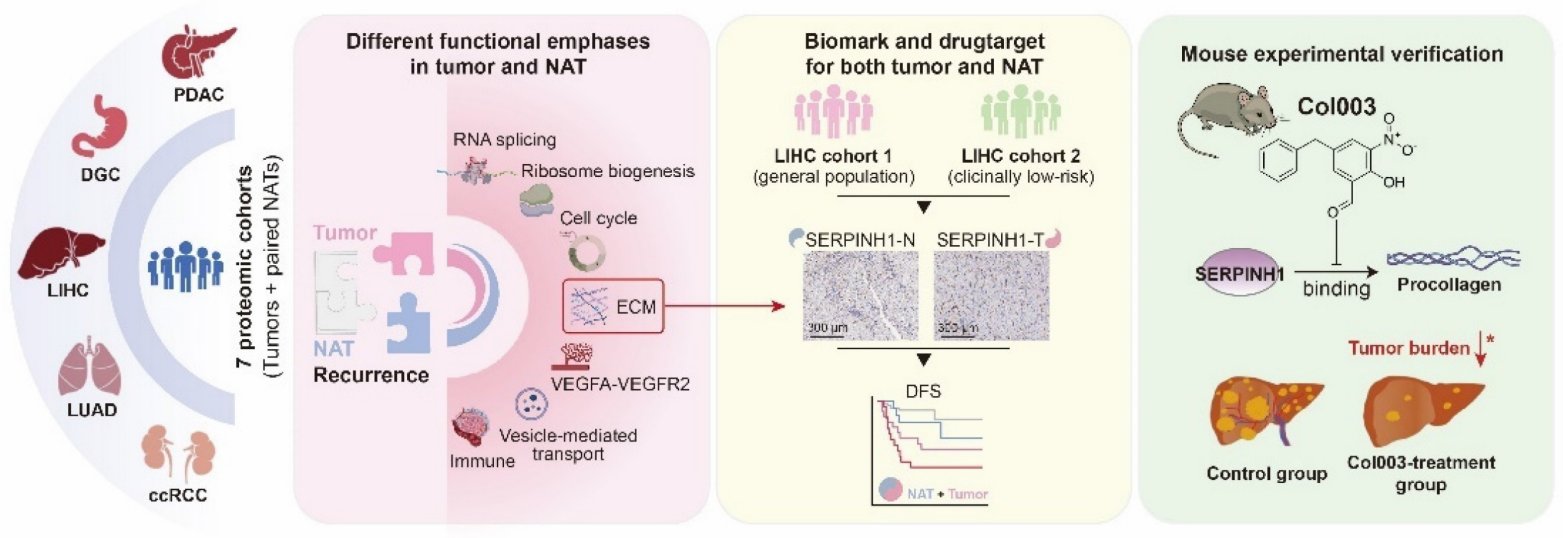
Study overview and major findings.

In summary (Figure 8), this study utilized seven proteomic datasets to demonstrate that the molecular characteristics of NATs are independent prognostic factors, complementing clinical indicators and tumor features, and playing an essential role in recurrence. It was proposed that attention should be paid to patients with unfavorable characteristics in NATs and treated accordingly. Taking liver cancer as an example, SERPINH1 was identified as an effective biomarker and drug target applicable to both tumors and NATs. This finding has been validated through independent immunohistochemistry cohorts and animal experiments, offering new insights for tumor recurrence research and clinical management.

## Supplementary Material

Supplementary figures.

Supplementary tables.

## Abbreviations

HCC: hepatocellular carcinoma
PDAC: pancreatic ductal adenocarcinoma
DGC: diffuse gastric cancer
LUAD: lung adenocarcinoma
ccRCC: clear cell renal cell carcinoma
NAT: non-tumor adjacent tissue
MVI: microvascular invasion
AFP: alpha-fetoprotein
CTC: circulating tumor cells
ctDNA: circulating tumor DNA
TACE: transarterial chemoembolization
HR: hazard ratio
RRP: recurrence-related protein
ES: enrichment score
GSEA: Gene set enrichment analysis
IHC: immunohistochemistry
TMA: Tissue microarray

## References

[1] Siegel RL, Giaquinto AN, Jemal A (2024). Cancer statistics, 2024. CA: A Cancer Journal for Clinicians.

[2] Bray F, Laversanne M, Sung H, Ferlay J, Siegel RL, Soerjomataram I (2024). Global cancer statistics 2022: GLOBOCAN estimates of incidence and mortality worldwide for 36 cancers in 185 countries. CA: A Cancer Journal for Clinicians.

[3] Vogel A, Meyer T, Sapisochin G, Salem R, Saborowski A (2022). Hepatocellular carcinoma. The Lancet.

[4] Finn RS, Zhu AX (2020). Evolution of Systemic Therapy for Hepatocellular Carcinoma. Hepatology.

[5] Llovet JM, Pinyol R, Kelley RK, El-Khoueiry A, Reeves HL, Wang XW (2022). Molecular pathogenesis and systemic therapies for hepatocellular carcinoma. Nature Cancer.

[6] Imamura H, Matsuyama Y, Tanaka E, Ohkubo T, Hasegawa K, Miyagawa S (2003). Risk factors contributing to early and late phase intrahepatic recurrence of hepatocellular carcinoma after hepatectomy. Journal of Hepatology.

[7] Yao LQ, Chen ZL, Feng ZH, Diao YK, Li C, Sun HY (2022). Clinical Features of Recurrence After Hepatic Resection for Early-Stage Hepatocellular Carcinoma and Long-Term Survival Outcomes of Patients with Recurrence: A Multi-institutional Analysis. Ann Surg Oncol.

[8] Sun Y-F, Xu Y, Yang X-R, Guo W, Zhang X, Qiu S-J (2013). Circulating stem cell-like epithelial cell adhesion molecule-positive tumor cells indicate poor prognosis of hepatocellular carcinoma after curative resection. Hepatology.

[9] Zhu GQ, Liu WR, Tang Z, Qu WF, Fang Y, Jiang XF (2022). Serial circulating tumor DNA to predict early recurrence in patients with hepatocellular carcinoma: a prospective study. Mol Oncol.

[10] Wang J, Huang A, Wang Y-P, Yin Y, Fu P-Y, Zhang X (2020). Circulating tumor DNA correlates with microvascular invasion and predicts tumor recurrence of hepatocellular carcinoma. Annals of Translational Medicine.

[11] Ye QH, Qin LX, Forgues M, He P, Kim JW, Peng AC (2003). Predicting hepatitis B virus-positive metastatic hepatocellular carcinomas using gene expression profiling and supervised machine learning. Nat Med.

[12] Roessler S, Jia HL, Budhu A, Forgues M, Ye QH, Lee JS (2010). A unique metastasis gene signature enables prediction of tumor relapse in early-stage hepatocellular carcinoma patients. Cancer Res.

[13] Jiang Y, Sun A, Zhao Y, Ying W, Sun H, Yang X (2019). Proteomics identifies new therapeutic targets of early-stage hepatocellular carcinoma. Nature.

[14] Gao Q, Zhu H, Dong L, Shi W, Chen R, Song Z (2019). Integrated Proteogenomic Characterization of HBV-Related Hepatocellular Carcinoma. Cell.

[15] Xing X, Hu E, Ouyang J, Zhong X, Wang F, Liu K (2023). Integrated omics landscape of hepatocellular carcinoma suggests proteomic subtypes for precision therapy. Cell Reports Medicine.

[16] Wang Z, Ren Z, Chen Y, Hu J, Yang G, Yu L (2018). Adjuvant Transarterial Chemoembolization for HBV-Related Hepatocellular Carcinoma After Resection: A Randomized Controlled Study. Clinical Cancer Research.

[17] Chen X, Zhang B, Yin X, Ren Z, Qiu S, Zhou J (2013). Lipiodolized transarterial chemoembolization in hepatocellular carcinoma patients after curative resection. Journal of Cancer Research and Clinical Oncology.

[18] Gao Y, Bado I, Wang H, Zhang W, Rosen JM, Zhang XHF (2019). Metastasis Organotropism: Redefining the Congenial Soil. Developmental Cell.

[19] Paget S (1889). THE DISTRIBUTION OF SECONDARY GROWTHS IN CANCER OF THE BREAST. The Lancet.

[20] Hoshida Y, Villanueva A, Kobayashi M, Peix J, Chiang DY, Camargo A (2008). Gene expression in fixed tissues and outcome in hepatocellular carcinoma. N Engl J Med.

[21] Tsuchiya M, Parker JS, Kono H, Matsuda M, Fujii H, Rusyn I (2010). Gene expression in nontumoral liver tissue and recurrence-free survival in hepatitis C virus-positive hepatocellular carcinoma. Mol Cancer.

[22] Gu Y, Guo Y, Gao N, Fang Y, Xu C, Hu G (2022). The proteomic characterization of the peritumor microenvironment in human hepatocellular carcinoma. Oncogene.

[23] Zhu X-D, Zhang J-B, Zhuang P-Y, Zhu H-G, Zhang W, Xiong Y-Q (2008). High Expression of Macrophage Colony-Stimulating Factor in Peritumoral Liver Tissue Is Associated With Poor Survival After Curative Resection of Hepatocellular Carcinoma. Journal of Clinical Oncology.

[24] Zhou H, Huang H, Shi J, Zhao Y, Dong Q, Jia H (2010). Prognostic value of interleukin 2 and interleukin 15 in peritumoral hepatic tissues for patients with hepatitis B-related hepatocellular carcinoma after curative resection. Gut.

[25] Qin S, Chen M, Cheng AL, Kaseb AO, Kudo M, Lee HC (2023). Atezolizumab plus bevacizumab versus active surveillance in patients with resected or ablated high-risk hepatocellular carcinoma (IMbrave050): a randomised, open-label, multicentre, phase 3 trial. Lancet.

[26] Wang K, Xiang Y-J, Yu H-M, Cheng Y-Q, Liu Z-H, Qin Y-Y (2024). Adjuvant sintilimab in resected high-risk hepatocellular carcinoma: a randomized, controlled, phase 2 trial. Nature Medicine.

[27] Tong Y, Sun M, Chen L, Wang Y, Li Y, Li L (2022). Proteogenomic insights into the biology and treatment of pancreatic ductal adenocarcinoma. Journal of Hematology & Oncology.

[28] Shi W, Wang Y, Xu C, Li Y, Ge S, Bai B (2023). Multilevel proteomic analyses reveal molecular diversity between diffuse-type and intestinal-type gastric cancer. Nature Communications.

[29] Xu J-Y, Zhang C, Wang X, Zhai L, Ma Y, Mao Y (2020). Integrative Proteomic Characterization of Human Lung Adenocarcinoma. Cell.

[30] Qu Y, Feng J, Wu X, Bai L, Xu W, Zhu L (2022). A proteogenomic analysis of clear cell renal cell carcinoma in a Chinese population. Nature Communications.

[31] Xu S, Xu H, Wang W, Li S, Li H, Li T (2019). The role of collagen in cancer: from bench to bedside. Journal of Translational Medicine.

[32] Ohta Y, Fujii M, Takahashi S, Takano A, Nanki K, Matano M (2022). Cell-matrix interface regulates dormancy in human colon cancer stem cells. Nature.

[33] Papanicolaou M, Parker AL, Yam M, Filipe EC, Wu SZ, Chitty JL (2022). Temporal profiling of the breast tumour microenvironment reveals collagen XII as a driver of metastasis. Nature Communications.

[34] Bonnans C, Chou J, Werb Z (2014). Remodelling the extracellular matrix in development and disease. Nature Reviews Molecular Cell Biology.

[35] Taguchi T, Razzaque MS (2007). The collagen-specific molecular chaperone HSP47: is there a role in fibrosis?. Trends in Molecular Medicine.

[36] Cardoso-Moreira M, Halbert J, Valloton D, Velten B, Chen C, Shao Y (2019). Gene expression across mammalian organ development. Nature.

[37] Popescu D-M, Botting RA, Stephenson E, Green K, Webb S, Jardine L (2019). Decoding human fetal liver haematopoiesis. Nature.

[38] Ma L, Wang L, Khatib SA, Chang C-W, Heinrich S, Dominguez DA (2021). Single-cell atlas of tumor cell evolution in response to therapy in hepatocellular carcinoma and intrahepatic cholangiocarcinoma. Journal of Hepatology.

[39] Bellaye P-S, Burgy O, Bonniaud P, Kolb M (2021). HSP47: a potential target for fibrotic diseases and implications for therapy. Expert Opinion on Therapeutic Targets.

[40] Uehara T, Pogribny IP, Rusyn I (2014). The DEN and CCl4-Induced Mouse Model of Fibrosis and Inflammation-Associated Hepatocellular Carcinoma. Current Protocols.

[41] Zhang J, Lin X-T, Fang L, Xie C-M (2023). In vivo analysis of FBXO45-mediated fibrosis and liver tumorigenesis in a chemically induced mouse model of hepatocellular carcinoma. STAR Protocols.

[42] Ito S, Ogawa K, Takeuchi K, Takagi M, Yoshida M, Hirokawa T (2017). A small-molecule compound inhibits a collagen-specific molecular chaperone and could represent a potential remedy for fibrosis. Journal of Biological Chemistry.

[43] Bruix J, Takayama T, Mazzaferro V, Chau G-Y, Yang J, Kudo M (2015). Adjuvant sorafenib for hepatocellular carcinoma after resection or ablation (STORM): a phase 3, randomised, double-blind, placebo-controlled trial. The Lancet Oncology.

[44] Liao H, Du J, Wang H, Lan T, Peng J, Wu Z (2021). Integrated proteogenomic analysis revealed the metabolic heterogeneity in noncancerous liver tissues of patients with hepatocellular carcinoma. Journal of Hematology & Oncology.

[45] Zhu H, Lin Y, Lu D, Wang S, Liu Y, Dong L (2023). Proteomics of adjacent-to-tumor samples uncovers clinically relevant biological events in hepatocellular carcinoma. National Science Review.

[46] Gong J, Li R, Chen Y, Zhuo Z, Chen S, Cao J (2021). HCC subtypes based on the activity changes of immunologic and hallmark gene sets in tumor and nontumor tissues. Briefings in Bioinformatics.

[47] Lewis JA (1999). Statistical principles for clinical trials (ICH E9): an introductory note on an international guideline. Stat Med.

[48] Rocnik E, Chow LH, Pickering JG (2000). Heat shock protein 47 is expressed in fibrous regions of human atheroma and Is regulated by growth factors and oxidized low-density lipoprotein. Circulation.

[49] Ito S, Nagata K (2021). Quality Control of Procollagen in Cells. Annual Review of Biochemistry.

[50] Tsan MF, Gao B (2004). Heat shock protein and innate immunity. Cell Mol Immunol.

[51] Xiong G, Chen J, Zhang G, Wang S, Kawasaki K, Zhu J (2020). Hsp47 promotes cancer metastasis by enhancing collagen-dependent cancer cell-platelet interaction. Proceedings of the National Academy of Sciences.

[52] Thienel M, Müller-Reif JB, Zhang Z, Ehreiser V, Huth J, Shchurovska K (2023). Immobility-associated thromboprotection is conserved across mammalian species from bear to human. Science.

[53] Wang Y, Gu W, Wen W, Zhang X (2022). SERPINH1 is a Potential Prognostic Biomarker and Correlated With Immune Infiltration: A Pan-Cancer Analysis. Frontiers in Genetics.

[54] Lawitz EJ, Shevell DE, Tirucherai GS, Du S, Chen W, Kavita U (2021). BMS-986263 in patients with advanced hepatic fibrosis: 36-week results from a randomized, placebo-controlled phase 2 trial. Hepatology.

