# Supplementary material for: Integrated analysis of tumor and adjacent non-tumor proteomic data reveals SERPINH1 as a recurrence biomarker and drug target in hepatocellular carcinoma
Source: Int J Biol Sci. 2024 Sep 23;20(13):5191–207. doi: 10.7150/ijbs.99734 (PMC11489177; doi:10.7150/ijbs.99734)
Supplement: Supplementary file 1 — Supplementary figures. [file ijbsv20p5191s1.pdf]

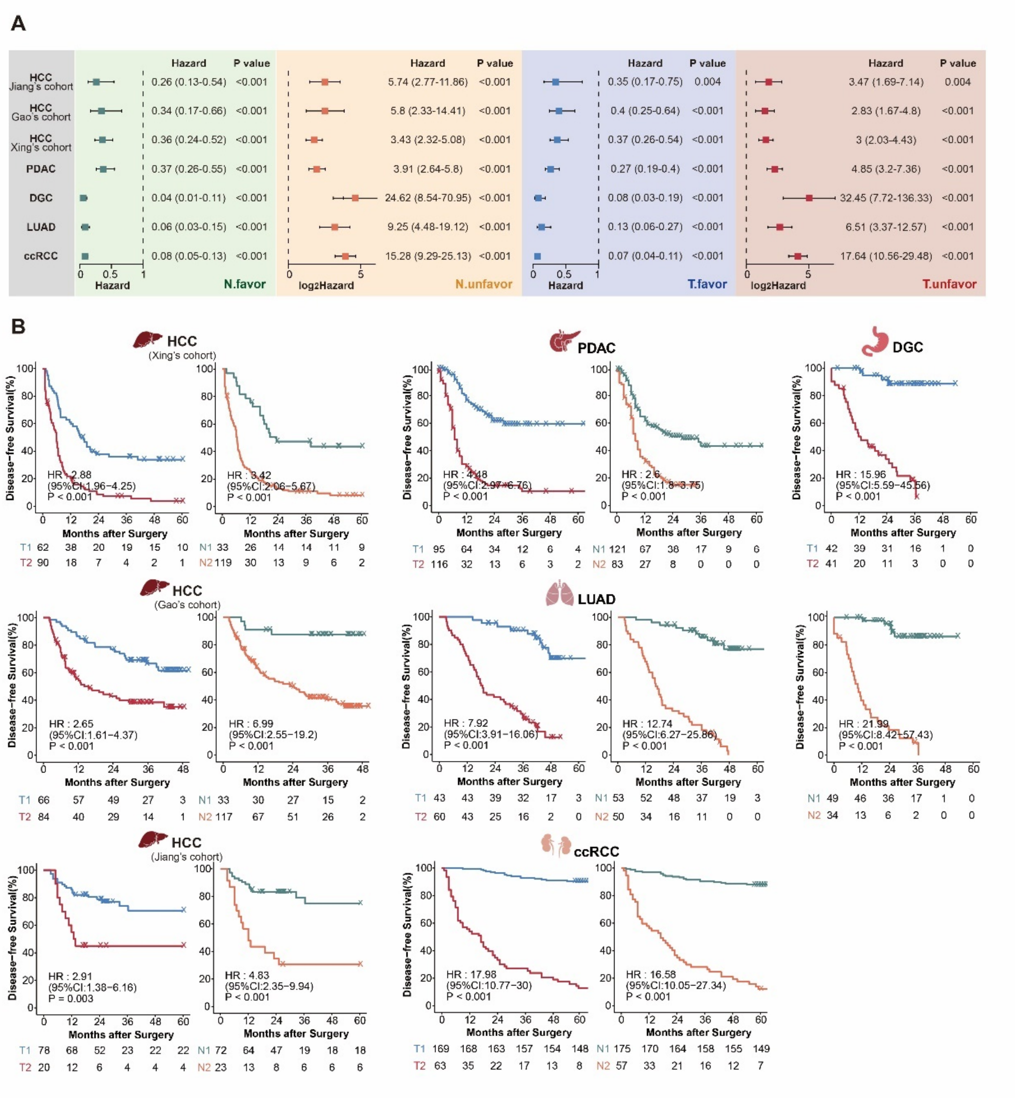

Figure S1. Recurrence-subgroups based on tumors or NATs can reliably predict recurrence. (A) The forest plots showed that four gene sets had stable predictive efficacy in multiple cancers. (B) Kaplan-Meier curves showed that DFS scores of tumors or NATs could divide patients into two groups with significant recurrence differences.

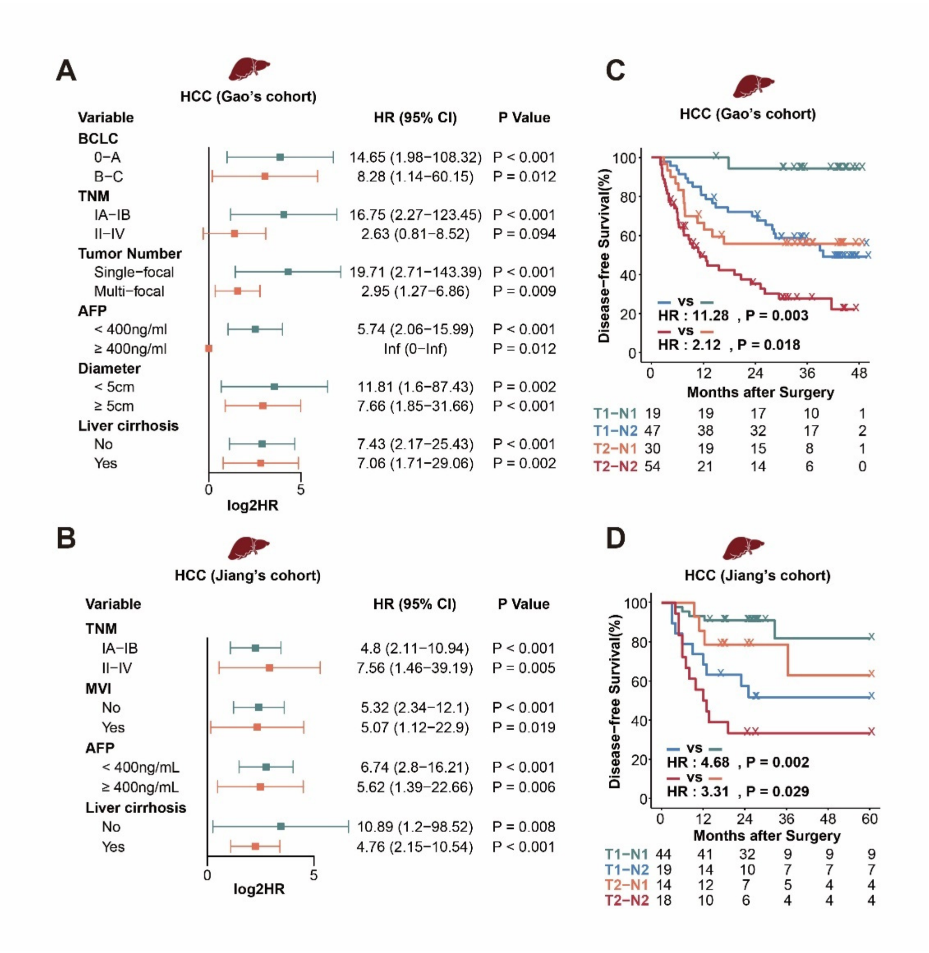

Figure S2. The NAT subgroups complement the clinical and molecular features of HCC for prognostic assessment. (A and B), NAT subgroups can stably stratify the recurrence risk for patients with high and low recurrence risk defined by clinical indicators and staging. (C and D), Integrating tumor and NAT recurrence subgroups can achieve a more accurate recurrence risk assessment.

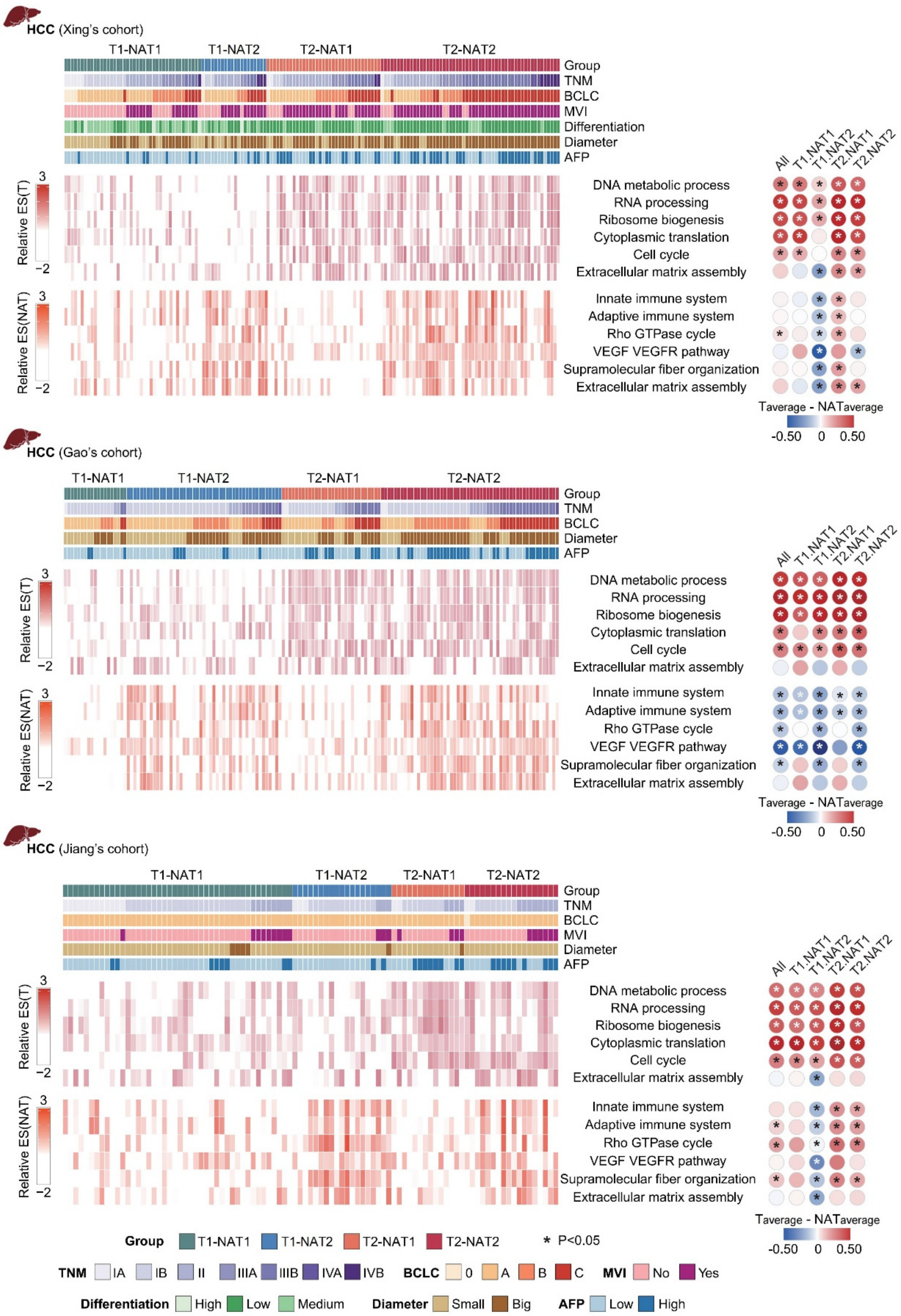

Figure S3. Differences in recurrence-related pathways between tumors and matched NATs. The color of the heat map on the right represents the difference between the mean values of tumor and matched NAT tissues. The red circle indicates the expression level of tumor is higher than that of NAT tissues. * represents a statistically significant difference. Statistical test: Wilcoxon. * P<0.05.

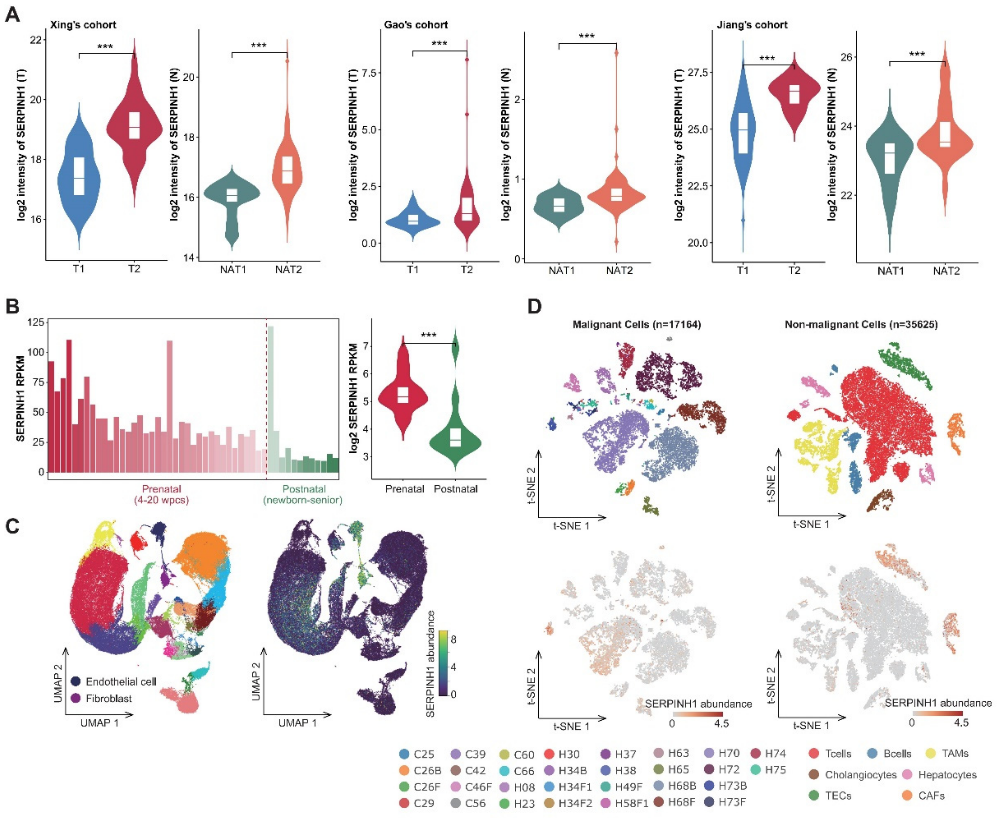

Figure S4. Expression characteristics of SERPINH1. (A) Compared with the T1 and NAT1 subgroups, the expression level of SERPINH1 is higher in the T2 and NAT2 subgroups. (B) The expression of SERPINH1 was significantly higher in the human fetal liver than in postnatal liver tissues. (C) SERPINH1 is mainly localized in vascular endothelial cells and fibroblasts, whose cell proportions significantly decrease during human fetal liver development. (D) In hepatocellular carcinoma samples, SERPINH1 is mainly localized in vascular endothelial cells and fibroblasts.

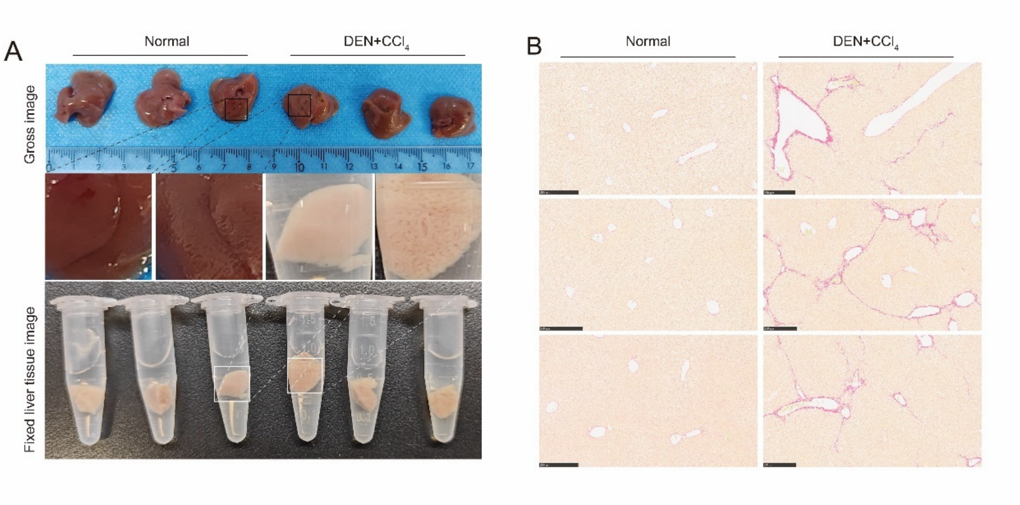

Figure S5. Ten-week administration of CCl_4-induced liver fibrosis in mice. After ten weeks of establishing mouse liver cancer models induced by DEN and CCl_4, liver tissues from both model mice and normal mice were randomly selected and fixed with 4% PFA for HE staining and Sirius red staining. (A) Gross image and fixed liver tissue image were shown. (B) Representative images of mouse liver tissue sections stained with Sirius Red were shown (Scale bar, 250 μm).

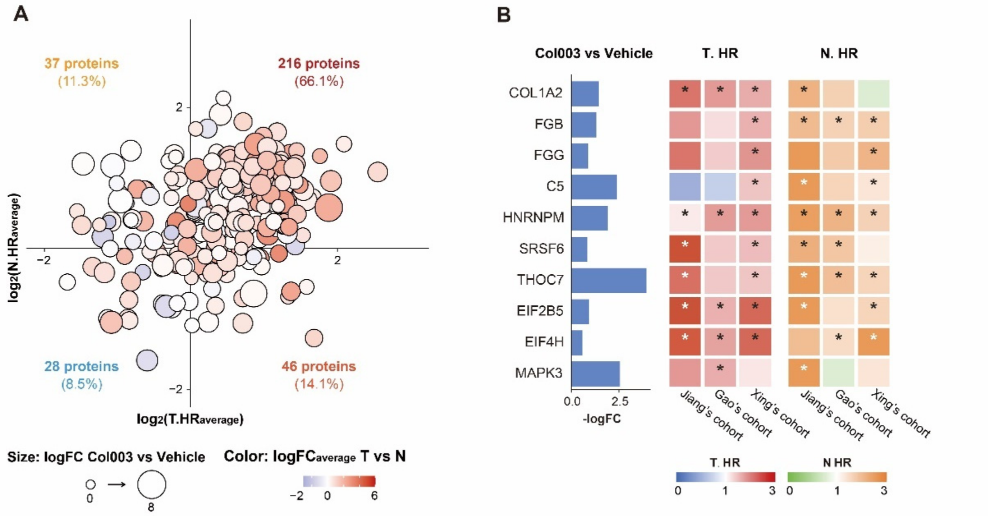

Figure S6. Prognostic characteristics of significantly down-regulated genes in the Col003-treated group. (A) The majority of the significantly downregulated proteins in the treated group were unfavorable in the tumors or NATs, accounting for approximately 91.5%. The X value corresponds to the average HR of tumors, and the Y value corresponds to the average HR of NATs. (B) Representative proteins were significantly down-regulated in the Col003-treated group compared to the control group.

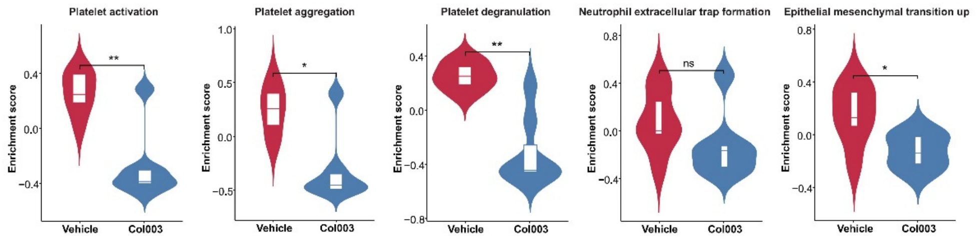

Figure S7. Difference analysis of platelet, neutrophil, and epithelial-mesenchymal transition-related pathways between the control group and Col003-treated group. Statistical test: Wilcoxon. * <0.05, **P <0.01, ***P <0.001.

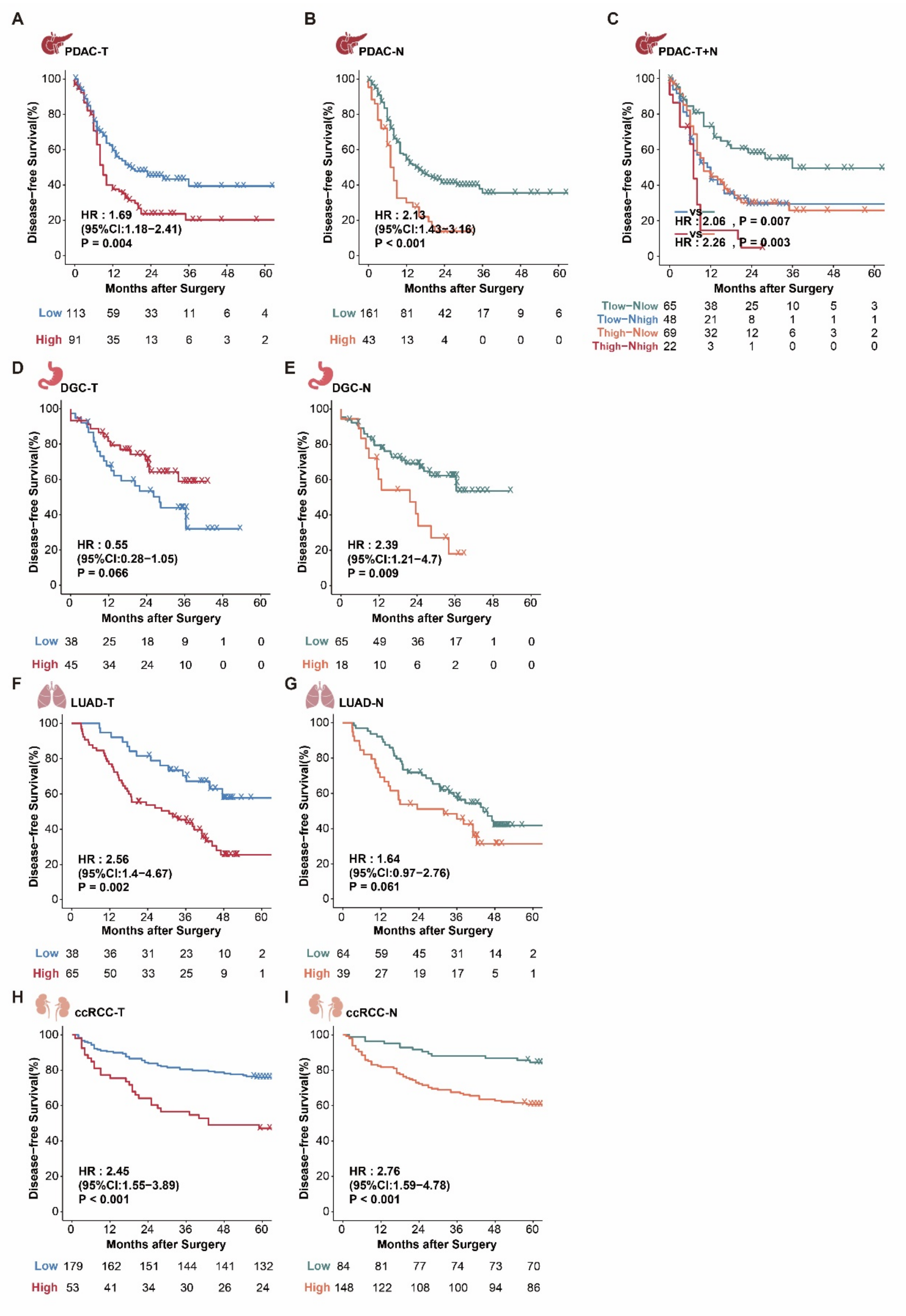

Figure S8. SERPINH1 has stable recurrence prediction ability in various tumors.

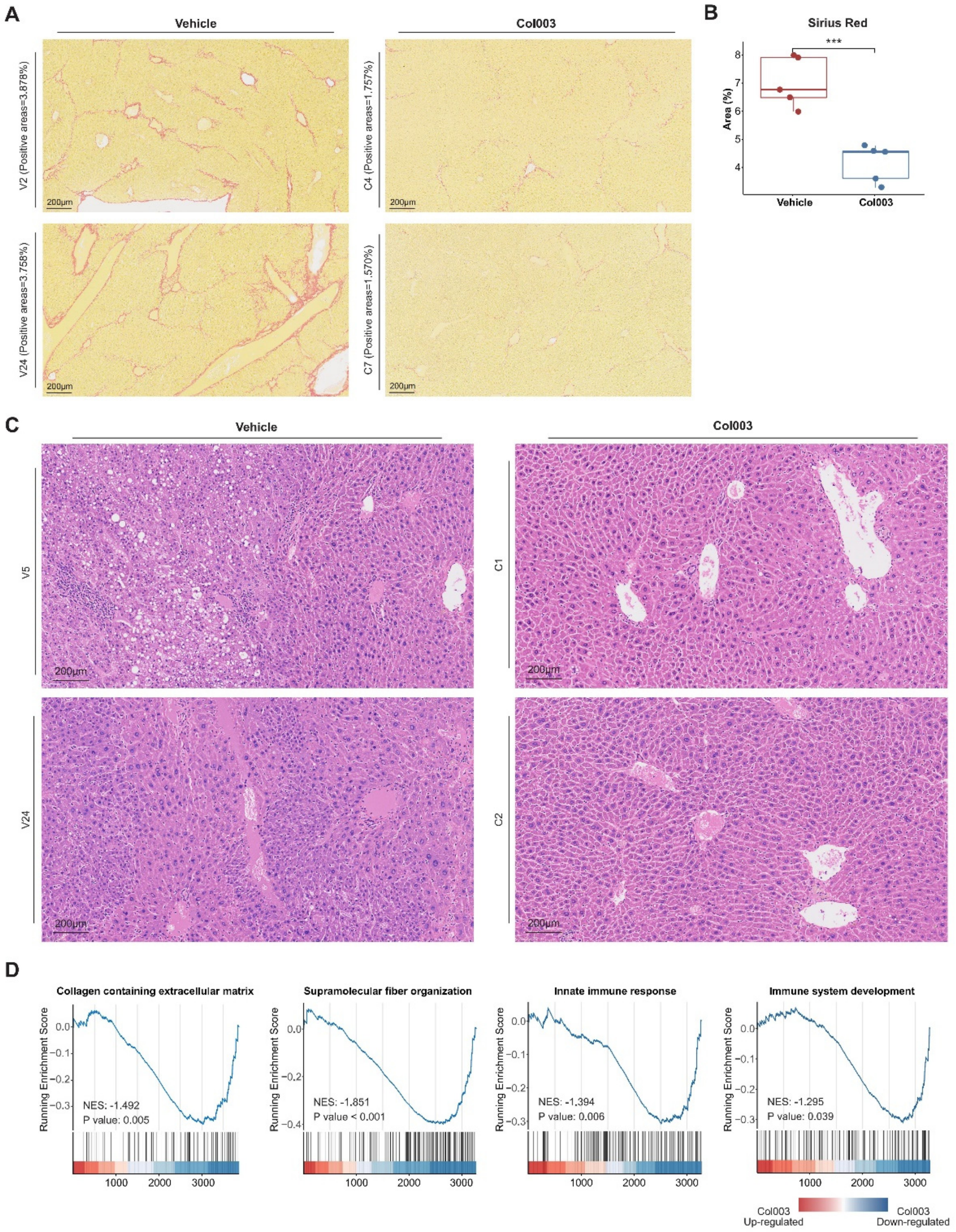

Figure S9. Effects of Col003 on liver fibrosis and hepatitis. (A) Representative images of histological sections stained with Sirius Red in the control group and the Col003-treated group. (B) Difference analysis of Sirius red staining area between the control group and the Col003-treated group. (C) Representative areas of H&E staining in the control group and the Col003-treated group. (D) GSEA analysis of immune and fibrosis-related pathways between the control group and the Col003-treated group.
